# Comparison of the renal outcomes of novel antidiabetic agents in patients with type 2 diabetes with chronic kidney disease: A systematic review and network meta‐analysis of randomized controlled trials

**DOI:** 10.1111/dom.70224

**Published:** 2025-10-28

**Authors:** Rong Lin, Chia‐Li Hsu, Ming‐Chieh Shih, Kuo‐Liong Chien, Hon‐Yen Wu

**Affiliations:** ^1^ Department of Internal Medicine Far Eastern Memorial Hospital New Taipei City Taiwan; ^2^ Ministry of Health and Welfare Shuang‐Ho Hospital New Taipei City Taiwan; ^3^ School of Medicine National Tsing Hua University Hsinchu Taiwan; ^4^ Institute of Epidemiology and Preventive Medicine National Taiwan University Taipei Taiwan; ^5^ Department of Internal Medicine National Taiwan University Hospital and College of Medicine Taipei Taiwan; ^6^ School of Medicine, College of Medicine National Yang Ming Chiao Tung University Taipei Taiwan

**Keywords:** antidiabetic drug, diabetic nephropathy, DPP‐IV inhibitor, GLP‐1 analogue, network meta‐analysis, SGLT2 inhibitor

## Abstract

**Aims:**

To investigate the renal outcomes of dipeptidyl peptidase 4 (DPP‐4) inhibitors, glucagon‐like peptide‐1 (GLP‐1) receptor agonists, and sodium‐glucose transport protein‐2 (SGLT‐2) inhibitors in patients with type 2 diabetes mellitus (T2DM) with chronic renal disease (CKD).

**Materials and Methods:**

PubMed, Embase, Cochrane CENTRAL, and ClinicalTrials.gov were searched through July 2025 for randomized controlled trials with ≥24 weeks of follow‐up in patients with T2DM and CKD. Outcomes included composite renal outcome, estimated glomerular filtration rate (eGFR), and urinary albumin‐to‐creatinine ratio (UACR). A network meta‐analysis was conducted, and the certainty of evidence was assessed with the Grading of Recommendations Assessment, Development, and Evaluation used to evaluate evidence certainty (GRADE).

**Results:**

Twenty RCTs enrolling 80,670 participants were included. Compared with placebo, several agents significantly reduced composite renal outcomes, with dapagliflozin 10 mg showing the greatest efficacy (OR 0.55, 95% CI 0.42–0.72; high‐certainty evidence), followed by canagliflozin, empagliflozin, efpeglenatide, sotagliflozin 400 mg, semaglutide, and dulaglutide 1.5 mg. Canagliflozin 100–300 mg significantly reduced UACR, whereas dapagliflozin had no effect. None of the novel antidiabetic agents significantly altered eGFR. Certainty of evidence ranged from high for placebo‐controlled comparisons to low or very low for indirect estimates.

**Conclusions:**

In patients with T2DM and CKD, SGLT2 inhibitors provide the most consistent renal protection, while GLP‐1 receptor agonists offer additional but variable benefits. Dapagliflozin showed the greatest efficacy, and canagliflozin most strongly reduced albuminuria, highlighting meaningful heterogeneity across agents. DPP‐4 inhibitors conferred no renal benefit. Overall, evidence from placebo‐controlled trials was robust, whereas certainty was lower for indirect estimates, highlighting the need for drug‐specific evaluation in clinical practice.

## 1 INTRODUCTION

Diabetic kidney disease (DKD) was one of the leading causes of end‐stage renal disease globally.^1^ Prevention of the decline of renal function is important in patients with type 2 diabetes mellitus (T2DM) or chronic kidney disease (CKD).^2^ Traditionally, renin‐angiotensin system blockers have been used to reduce proteinuria and postpone CKD progression.^3^ Recently, novel antidiabetic agents have been shown not only to lower blood sugar levels but also to provide renoprotective effects in diabetic kidney disease. These include dipeptidyl peptidase 4 (DDP‐4) inhibitors,^4^, ^5^ glucagon‐like peptide‐1 (GLP‐1) agonists^6^ and sodium‐glucose co‐transporter‐2 (SGLT‐2) inhibitors.^7^, ^8^, ^9^, ^10^, ^11^, ^12^

The 2025 American Diabetes Association guidelines introduced significant updates based on recent evidence regarding the cardiorenal benefits of novel antidiabetic agents in patients with T2DM. These guidelines emphasize assessing whether patients have atherosclerotic cardiovascular disease and recommend medications that reduce cardiorenal risk.^13^ However, in clinical practice, when managing patients with both T2DM and CKD, it is not clear which class of medication should be prioritized or which specific agent is the most effective. While previous studies have compared different drug classes,^14^ current guidelines do not provide direct individual drug comparisons.

With the emergence of new clinical studies, we aimed to conduct a systematic review and network meta‐analysis to evaluate the renal outcomes of individual agents in these three drug classes in patients with T2DM and CKD.

## 2 MATERIALS AND METHODS

### 2.1 Protocol and registration

This systematic review and network meta‐analysis followed the Preferred Reporting Items for Systematic Reviews and Meta‐analyses (PRISMA‐NMA, Table S1). The protocol for this study was registered in the International Prospective Register of Systematic Reviews (PROSPERO; registration number CRD42024464258).

### 2.2 Data sources and search strategy

We systematically searched the electronic literature of PubMed, Embase, the Cochrane Central Register of Controlled Trials, and ClinicalTrials.gov according to a predefined protocol to identify relevant randomized controlled trials (RCTs) from inception up to July 2025 without language restriction but limited to adult humans. Details of the search terms are provided in the Data S1. Two additional studies were identified by manually searching the references of the included studies and other meta‐analyses.

### 2.3 Study selection

After removing duplicate studies, two reviewers (Rong Lin and Chia‐Li Hsu) independently screened the titles, abstracts, and full texts of the remaining studies to select possible trials based on our inclusion criteria: (1) randomized controlled trials (RCTs, parallel design but not cross‐over); (2) two treatment arms, in which any DPP‐4 inhibitor, GLP‐1 receptor agonist, or SGLT‐2 inhibitor was compared with placebo (or usual treatment) to assess kidney disease outcomes in patients with type 2 diabetes and CKD; (3) reporting outcomes in diabetic CKD subgroups; and (4) a study duration of at least 24 weeks. Exclusion criteria included: (1) RCTs on other types of diabetes (e.g., type 1 diabetes mellitus or gestational diabetes); and (2) post‐hoc analyses of RCTs, which were excluded because they are not pre‐specified in the trial protocols, are exploratory in nature, and may increase the risk of selective reporting bias.

### 2.4 Outcomes

Three outcomes were assessed in our study: (1) the change in estimated glomerular filtration rate (eGFR) is reported as the mean difference with a 95% confidence interval (CI), (2) the change in urine albumin‐creatinine ratio (UACR) is reported as the ratio of fold change with a 95% CI, and (3) differences in composite renal outcomes (doubling of creatinine levels, 30% reduction in GFR, acute kidney injury, end‐stage kidney disease, or renal death) are reported as odds ratios (ORs) with 95% CIs.

### 2.5 Data extraction

Two reviewers (Rong Lin and Chia‐Li Hsu) independently extracted the data according to a predefined protocol using data collection software (Covidence, Veritas Health Innovation, Melbourne, Australia; available at www.covidence.org). The data extracted included study characteristics (first author, year of publication, country of origin, inclusion criteria for each trial, and sample size), population (sex, age, duration of diabetes, glycated haemoglobin [HbA1c], eGFR, type of intervention, and outcomes of interest).

### 2.6 Risk of bias and evidence certainty assessment

The risk of bias was assessed by two reviewers (Rong Lin and Chia‐Li Hsu) using version 2 of the Cochrane risk of bias tool for randomized trials (RoB 2),^15^ which included random sequence generation, allocation concealment, blinding, missing outcome data, and selective reporting of results. Each domain was considered to have a low risk, some concerns, or high risk of bias.^16^

We also used the Grading of Recommendations Assessment, Development, and Evaluation (GRADE) method to evaluate the level of confidence in the evidence from direct, indirect, and network estimates for all outcomes. We also used an Excel sheet provided by the GRADE Working Group to simplify the complicated steps.^17^ According to this method, the certainty of evidence from RCTs initially starts as high but may be downgraded to moderate, low, or very low owing to factors such as risk of bias, indirectness, inconsistency, or intransitivity. Regarding indirectness, we evaluated whether there were significant differences between the ethnic groups, intervention measures, control measures, and expected results reported in the studies and the actual application between different papers; if there were differences, they were downgraded. The Population, Intervention, Comparator, and Outcome (PICO) elements of the included studies were compared using our study protocol.^18^ We used a side‐splitting approach to analyse inconsistency.^19^ Publication bias was detected using funnel plots and Egger's test.^20^ When evaluating RCTs, these factors must be considered.^21^, ^22^, ^23^ To judge the intransitivity, we evaluated the indirect part of patient characteristics, differing co‐interventions, differing extents to which interventions of interest were optimally administered, differing comparators, and differences in outcome measurements, and considered downgrading according to severity.

All evaluations were performed by two reviewers (Rong Lin and Chia‐Li Hsu); if there was any disagreement, a third reviewer (Hon‐Yen Wu) was involved.

### 2.7 Data synthesis and statistical analysis

#### 2.7.1 Network meta‐analysis

We generated a network diagram for each outcome of interest to inspect the connectivity of the network and identify possible weak spots for transitivity. We performed random effects network meta‐analyses under the frequentist paradigm using the R netmeta package.^24^ We opted for different effect measures for each outcome: for the composite renal outcome, which is binary, we used the odds ratio as an effect measure; for UACR, since the fold change is more commonly used for clinical evaluation, we opted for the ratio of means as an effect measure; and for eGFR, we used the mean difference as an effect measure. P‐scores, which are frequentist analogs of the surface under the cumulative ranking curve, were used to rank each treatment.^25^

#### 2.7.2 Exploring inconsistency

Inconsistencies were evaluated for each contrast, when permitted by the network design, using a symmetric side‐splitting model.^26^ Network meta‐analysis was performed using the R Netmeta package (version 3.2.0, updated by July 14, 2025).

## 3 RESULTS

### 3.1 Description of included studies

The PRISMA flow diagram shows the study selection process (Figure S1). We searched 1436 articles initially, and two articles were identified through references from other sources. A total of 208 articles were removed as duplicate publications, excluding studies based on the title, abstract, and full‐text review. Finally, 20 RCTs^5^, ^10^, ^12^, ^27^, ^28^, ^29^, ^30^, ^31^, ^32^, ^33^, ^34^, ^35^, ^36^, ^37^, ^38^, ^39^, ^40^, ^41^, ^42^, ^43^, ^44^ were eligible for inclusion, which enrolled 80,670 participants with both T2DM and CKD.

The baseline characteristics of included RCTs are summarized in Table 1. The sample size ranged from 40 to 14 752, with the follow‐up durations between 24 and 282 weeks (median 104 weeks). The age of participants ranged from 61.2 to 68.5 years, with 21% to 48% being women. The duration of T2DM ranged from 10.6 to 19.6 years, baseline HbA1c ranged from 7.6% to 8.9%, and baseline eGFR ranged from 42.0 to 56.1 mL/min/1.73 m^2^. Continuous variables are presented as mean ± SD or median (interquartile range, IQR), according to the original report of each trial. When dispersion measures were not available, only central tendency values are shown.

**TABLE 1:** Baseline characteristics of the included studies.

Author, year (Study)	Country	Inclusion criteria	Total No.	T2DM duration (years), mean ± SD*	BMI (kg/m^2^), mean ± SD*	HbA1c (%), mean ± SD*	Baseline eGFR (mL/min/1.73 m^2^), mean ± SD*	Baseline UACR (mg/g), median (IQR)*	Age (years), mean ± SD*	Female (%)	Follow‐up (wks)	Treatment/No.	Control/No.
DDP4i													
Rosenstock 2019 (CARMELINA)	Multinational	Age ≥18 (Japan ≥20), HbA1c: 6.5–10.0% eGFR: 45–75 + UACR>200 mg/g or eGFR: 15–45	6979	14.8	31.4	7.9	54.6	162.0	65.9 ± 9.1	37.10%	115	Linagliptin/3494	Placebo/3485
Han 2018 (GUARD extension)	Korea	Age: 19–75, HbA1c: 7.0–11.0%, eGFR: 15–59	132	16	26.4	8.4	34.1	378.0	62.4	36%	52	Gemigliptin/66	Linagliptin/66
GLP1a													
Perkovic 2024 (FLOW)	Multinational	Age ≥18 (Japan ≥20), HbA1c: 6.5–10.0%; eGFR: 50–75 + UACR:300–5000 mg/g or eGFR: 25–45 + UACR:100–5000 mg/g	3533	17.4	32 ± 6.3	7.8 ± 1.3	47 ± 15.2	567.6	66.6 ± 9.0	30.3%	234	Semaglutide/1767	Placebo/1766
Tuttle 2021 (AWARD‐7 exploratory)	Multinational	Age ≥18, HbA1c: 7.5–10.5%, eGFR: 15–60	576	18.1 ± 8.75	32.5 ± 5.25	8.6 ± 0.97	38.3 ± 12.83	N/A	64.6 ± 8.6	47.70%	52	Dulaglutide (1.5 mg + 0.75 mg)/382	Insulin glargine/194
Gerstein (2019) (REWIND)	Multinational	Age ≥ 50, HbA1c ≦9.5%, eGFR: >15	9901	10.6	32.3	7.4 ± 1.1	76.9	209.77	66.2 ± 6.5	46.40%	282	Dulaglutide/4949	Placebo/4952
Holman 2017 (EXSCEL)	Multinational	Age ≥ 18, HbA1c: 6.5–10, eGFR≧30	14 752	12	31.7	8	76.3	N/A	62 ± 9.4	38.00%	167	Exenatide/7356	Placebo/7396
SGLT2i													
Sridhar 2024 (SCORED exploratory analysis)	Multinational	Age ≥ 18, HbA1c ≥ 7%, eGFR:25–60	10 584	N/A	31.8	8.3	45	N/A	68.3 ± 8.4	44.9%	69.6	Sotagliflozin/5292	Placebo/5292
Cherney 2023	Multinational	Age ≥ 18, HbA1c: 7–11%, eGFR:30–60	787	17.1 ± 9.0	32.4 ± 5.4	8.3 ± 1.0	45 ± 8.1	440.70 (124.3–2457.1)	69.5 ± 7.9	43.6%	52	Sotagliflozin (400 mg + 200 mg)/527	Placebo/260
Herrington 2023 (EMPA‐KIDNEY)	Multinational	Age ≥ 18, eGFR:20–45 or eGFR: 45–90 + UACR>200	6609	N/A	29.8	N/A	37.3	329.0	63.8	33.10%	104	Empagliflozin/3304	Placebo/3305
Wada 2022	Japan	Age ≧30, HbA1c: 6.5–12%, eGFR: 30–90, UACR: 300–5000	308	15.97 ± 8.81	26.9 ± 4.5	7.76 ± 1.07	55.7 ± 14.6	683.0 (420–1330)	62.5 ± 10.7	20.80%	104	Canagliflozin/154	Placebo/154
Bhatt 2021 (SCORED)	Multinational	Age: 18–85, HbA1c ≥6.5%, eGFR: 25–60	10 584	N/A	31.8	8.3	44.5	74.5	68.3 ± 8.4	44.90%	70	Sotagliflozin/5292	Placebo/5292
Cherney 2021	Multinational	Age ≧18, HbA1c: 7–11, eGFR: 15–30	277	19.6	31.6	8.3	23.9	115900.33	67.4	51.30%	52	Sotagliflozin (400 mg + 200 mg)/184	Placebo/93
Dagogo‐Jack 2021	Multinational	Age ≥ 40, HbA1c: 7.0–10.5%, eGFR: 30–60	1776	15.6	32.4	8.2	49.3	N/A	68.2	35.70%	260	Ertugliflozin/1178	Placebo/598
Gerstein 2021 (AMPLITUDE‐O)	Multinational	Age ≥ 18, HbA1c: 7–11%, eGFR:25–60	4076	15.4 ± 8.8	32.7 ± 6.2	8.9	72.4 ± 22.4	28.3 (9.7–114.2)	64.5 ± 8.2	33%	94	Efpeglenatide (4 mg + 6 mg)/2717	Placebo/1359
Wheeler 2021 (DAPA‐CKD subgroup)	Multinational	Age ≥ 18, eGFR: 25–75, UACR: 200–5000	4304	N/A	N/A	7.1	43.1	950.0	61.8	33%	125	Dapagliflozin/2152	Placebo/2152
Perkovic 2019 (CREDENCE)	Multinational	Age ≥ 30, HbA1c: 6.5–12 (Germany HbA1c: 6.5–10.5) eGFR: 30–90, UACR: 300–5000 mg/g	4401	15.8 ± 8.6	31.3 ± 6.2	8.3	56.2 ± 18.2	927 (463–1833)	63 ± 9.2	33.90%	137	Canagliflozin/2204	Placebo/2199
Pollock 2019 (DELIGHT)	Multinational	Age ≧18, HbA1c: 7.0–11.0%, eGFR: 25–75, UACR: 30–3500	461	17.9	30.5	8.4	49	263.74	64.5	30%	24	Dapagliflozin/145	Placebo/148
Fioretto 2018 (DERIVE)	Multinational	Age: 18–75, HbA1c: 7.0–11.0%, eGFR: 45–60	321	14.4	32.1	8.2 ± 1.08	53.5	26.26	65.8	43.30%	24	Dapagliflozin/160	Placebo/161
Takashima 2018	Japan	Age ≥ 18, HbA1c <10%, eGFR: 45–90, UACR: 30–2000 mg/g	40	N/A	25.2	7.4	56.3	149	65.1	43.00%	52	Canagliflozin/20	Placebo/20
Yale 2014	Multinational	Age ≧25, HbA1c: 7.0–10.5%, eGFR: 30–50	269	16.3 ± 8.5	33.0 ± 6.2	8.0 ± 0.9	39.4	256.8	68.5 ± 8.3	39.40%	52	Canagliflozin (300 mg + 100 mg)/ 179	Placebo/90

*Note*: Continuous variables are expressed as mean ± SD, except UACR as median (IQR); when dispersion measures were not reported, only mean or median values are shown (* indicates missing SD or IQR in original study).

Abbreviations: BMI, body mass index; DPPi, dipeptidyl peptidase‐4 inhibitors; eGFR, estimated glomerular filtration rate; GLP1, glucagon‐like peptide‐1; SGLT2i, sodium‐glucose co‐transporter‐2 inhibitors; T2DM, type 2 diabetes mellitus; UACR, urine albumin‐creatinine ratio.

The included RCTs were published between 2014 and 2025, and three main categories of novel antidiabetic agents were identified: SGLT2 inhibitors (14 RCTs), GLP‐1 receptor agonists (4 RCTs), and DPP‐4 inhibitors (2 RCTs). Table 1 presents the baseline design and population characteristics of these studies, while Table S2 provides detailed information on the interventions, renal outcomes, and main findings of each trial. Together, these tables offer a comprehensive overview of both the study populations and the renal effects of novel antidiabetic agents, serving as the foundation for the subsequent pooled and network meta‐analyses.

### 3.2 Risk of bias

Quality assessment conducted using the ROB 2 is presented in Figure S2. Each study is presented with the corresponding drug and trial designation. Overall, eight studies were judged to have a low risk of bias, two at high risk, while the remaining studies were classified as having some concerns of bias. The most common sources of bias were related to selective reporting, incomplete outcome data, and measurement of outcomes.

### 3.3 Certainty of evidence

The certainty of evidence, assessed using the GRADE framework, varied across outcomes (Tables S3–S5). For composite renal outcomes, certainty ranged from high to very low, with several placebo‐controlled comparisons (e.g., dapagliflozin, canagliflozin, ertugliflozin, semaglutide vs. placebo) rated as high certainty, while indirect and head‐to‐head comparisons were frequently downgraded. For eGFR change, most placebo‐controlled comparisons were of moderate certainty, whereas head‐to‐head estimates were downgraded to low or very low certainty. For UACR, evidence was more variable, with dapagliflozin versus placebo supported by high certainty but many other comparisons downgraded to low or very low. Overall, placebo‐controlled comparisons provided the most robust and reliable evidence, whereas indirect and head‐to‐head comparisons remained limited in certainty.

### 3.4 Outcomes

#### 3.4.1 Composite renal outcome

**FIGURE 1:**
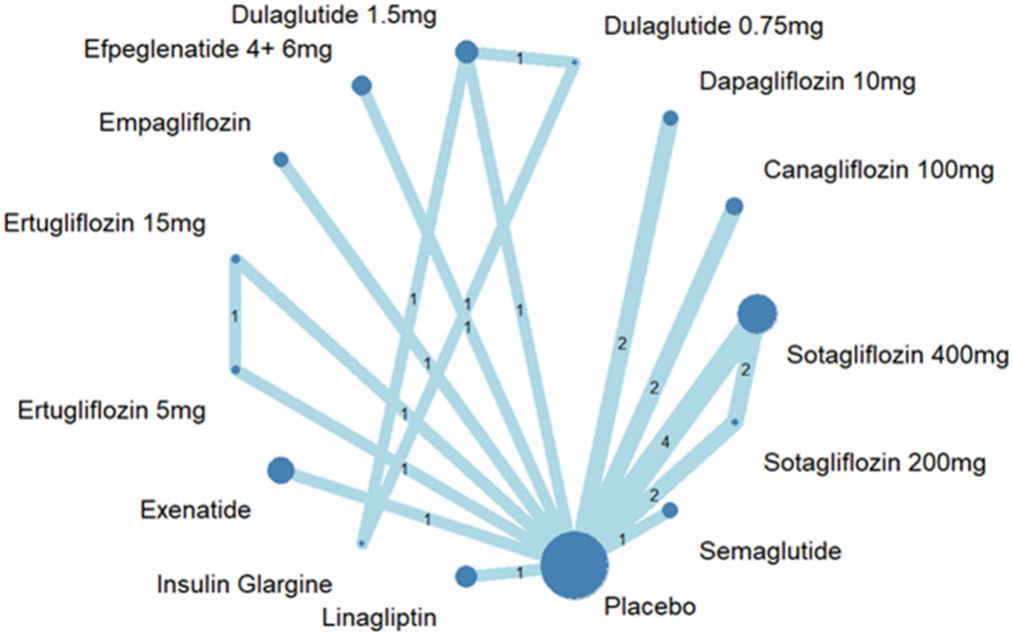
Network diagram of interventions for renal composite outcomes.

**FIGURE 2:**
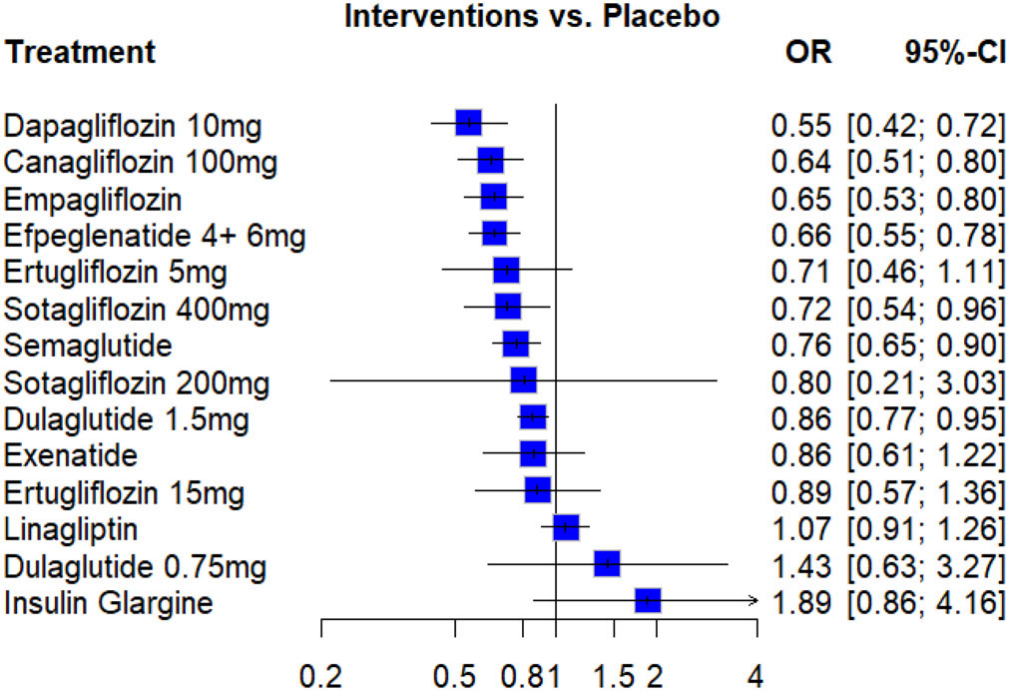
Forest plot of the network meta‐analysis comparing interventions with placebo for the renal composite outcome.

Figure 1 shows the network plot of the interventions,^10^, ^12^, ^28^, ^33^, ^34^, ^35^, ^36^, ^37^, ^38^, ^39^, ^40^, ^41^, ^42^, ^43^, ^44^ in the included studies that reported composite renal outcomes in patients with both T2DM and CKD. Table 2 summarizes the pooled relative effect estimates from the pairwise meta‐analysis presented in the upper‐right triangle. Figure 2 shows the forest plots for each direct treatment comparison. Several interventions significantly reduced the risk of composite renal outcomes compared with placebo; Dapagliflozin 10 mg demonstrated the greatest reduction in renal composite outcomes (odds ratio [OR] 0.55, 95% confidence interval [CI] 0.42–0.72), followed by canagliflozin 100 mg (OR 0.64, 95% CI 0.51–0.80), empagliflozin (OR 0.65, 95% CI 0.53–0.80), efpeglenatide 4 + 6 mg (OR 0.66, 95% CI 0.55–0.78), and sotagliflozin 400 mg (OR 0.72, 95% CI 0.54–0.96). Semaglutide and dulaglutide 1.5 mg also showed significant protective effects (OR 0.76, 95% CI 0.65–0.90; OR 0.86, 95% CI 0.77–0.95, respectively). In contrast, other agents such as linagliptin, exenatide, dulaglutide 0.75 mg, ertugliflozin (5 and 15 mg), and sotagliflozin 200 mg did not reach statistical significance, while insulin glargine was associated with a nonsignificant increased risk (OR 1.89, 95% CI 0.86–4.16).

**TABLE 2:** Estimated effects of each intervention on renal composite outcomes in patients with type 2 diabetes mellitus (T2DM) and chronic kidney disease (CKD) based on the network meta‐analysis (lower left triangle) and pairwise meta‐analysis (upper right triangle).

Dapagliflozin 10 mg											0.55 (0.12, 2.53)			
0.86 (0.61, 1.22)	Canagliflozin 100 mg										0.64 (0.18, 2.23)			
0.84 (0.61, 1.18)	0.98 (0.72, 1.33)	Empagliflozin												
0.84 (0.61, 1.15)	0.98 (0.73, 1.30)	1.00 (0.76, 1.30)	Efpeglenatide 4+ 6 mg											
0.77 (0.46, 1.29)	0.90 (0.55, 1.47)	0.92 (0.56, 1.49)	0.92 (0.57, 1.48)	Ertugliflozin 5 mg										
0.77 (0.52, 1.14)	0.89 (0.62, 1.29)	0.91 (0.64, 1.30)	0.91 (0.65, 1.29)	1.00 (0.58, 1.69)	Sotagliflozin 400 mg						0.72 (0.55, 0.94)			
**0.72 (0.53, 0.98)**	0.84 (0.64, 1.10)	0.86 (0.66, 1.11)	0.86 (0.68, 1.09)	0.94 (0.58, 1.50)	0.94 (0.67, 1.32)	Semaglutide								
0.69 (0.18, 2.65)	0.80 (0.21, 3.06)	0.81 (0.21, 3.11)	0.82 (0.21, 3.11)	0.89 (0.22, 3.60)	0.89 (0.24, 3.36)	0.95 (0.25, 3.61)	Sotagliflozin 200 mg				1.03 (0,43, 93.5)			
**0.64 (0.42, 0.99)**	0.75 (0.49, 1.13)	0.76 (0.51, 1.14)	0.76 (0.52, 1.13)	0.83 (0.47, 1.46)	0.84 (0.53, 1.32)	0.89 (0.61, 1.30)	0.93 (0.24, 3.68)	Exenatide						
**0.65 (0.49, 0.86)**	**0.75 (0.58, 0.96)**	**0.76 (0.61, 0.96)**	**0.77 (0.62, 0.94)**	0.83 (0.53, 1.32)	0.84 (0.61, 1.15)	0.89 (0.73, 1.08)	0.94 (0.25, 3.55)	1.00 (0.70, 1.44)	Dulaglutide 1.5 mg					
0.62 (0.38, 1.03)	0.72 (0.45, 1.18)	0.74 (0.46, 1.19)	0.74 (0.47, 1.18)	0.81 (0.51, 1.28)	0.81 (0.48, 1.37)	0.86 (0.54, 1.37)	0.91 (0.23, 3.66)	0.97 (0.56, 1.69)	0.97 (0.62, 1.51)	Ertugliflozin 15 mg				
**0.55 (0.42, 0.72)**	**0.64 (0.51, 0.80)**	**0.65 (0.53, 0.80)**	**0.66 (0.55, 0.78)**	0.71 (0.46, 1.11)	**0.72 (0.54, 0.96)**	**0.76 (0.65, 0.90)**	0.80 (0.21, 3.03)	0.86 (0.61, 1.22)	**0.86 (0.77, 0.95)**	0.89 (0.57, 1.36)	Placebo			
**0.51 (0.38, 0.70)**	**0.60 (0.45, 0.79)**	**0.61 (0.47, 0.79)**	**0.61 (0.48, 0.78)**	0.67 (0.42, 1.07)	0.67 (0.48, 0.94)	0.71 (0.57, 0.90)	0.75 (0.20, 2.85)	0.80 (0.55, 1.18)	0.80 (0.66, 0.97)	0.83 (0.52, 1.31)	0.93 (0.79, 1.10)	Linagliptin		
**0.39 (0.16, 0.92)**	0.45 (0.19, 1.05)	0.46 (0.20, 1.07)	0.46 (0.20, 1.07)	0.50 (0.20, 1.27)	0.50 (0.21, 1.20)	0.53 (0.23, 1.23)	0.56 (0.12, 2.67)	0.60 (0.25, 1.47)	0.60 (0.26, 1.35)	0.62 (0.24, 1.57)	0.70 (0.31, 1.59)	0.75 (0.32, 1.73)	Dulaglutide 0.75 mg	
**0.29 (0.13, 0.67)**	**0.34 (0.15, 0.77)**	**0.35 (0.15, 0.78)**	**0.35 (0.15, 0.78)**	**0.38 (0.15, 0.93)**	**0.38 (0.16, 0.88)**	**0.40 (0.18, 0.90)**	0.42 (0.09, 1.99)	0.45 (0.19, 1.08)	**0.45 (0.21, 0.99)**	0.47 (0.19, 1.15)	0.53 (0.24, 1.16)	0.57 (0.25, 1.27)	0.76 (0.38, 1.50)	Insulin Glargine

*Note*: Pooled effect estimates were presented as odds ratios (ORs) with 95% confidence intervals (CIs). Bold values indicate statistically significant differences. In the network meta‐analysis (lower left triangle), OR <1 suggests a favorable effect of column‐defining treatment on renal composite outcomes. In the pairwise meta‐analysis (upper right triangle), OR <1 indicates the benefit of the row‐defining treatment.

Treatment ranking based on P‐scores indicated that dapagliflozin 10 mg (P‐score 0.9205) ranked highest, followed by canagliflozin (0.8019), empagliflozin (0.782), and efpeglenatide (0.6817). In contrast, dulaglutide 0.75 mg (0.1818) and insulin glargine (0.0602) were ranked lowest (Table S6).

### 3.5 Change in eGFR

Nine studies reported changes in eGFR.^12^, ^27^, ^30^, ^31^, ^32^, ^36^, ^40^, ^42^, ^43^ These studies formed three disconnected subnetworks (Figure S3). Among them, one subnetwork contained all SGLT2 inhibitors from five studies, whereas DPP4 inhibitors and GLP1‐RA had one article each. To address this issue, we conducted a secondary analysis focusing on SGLT2 inhibitors, defined as an analysis restricted to one connected subnetwork to allow meaningful comparisons. As shown in the forest plot (Figure S4), none of the SGLT2 inhibitors demonstrated a statistically significant effect on the change in eGFR compared with placebo in patients with T2DM and CKD. The estimated mean differences (MD) in eGFR compared with placebo were −4.98 mL/min/1.73 m^2^ (95% CI –10.36 to 0.40) for canagliflozin 300 mg, −0.89 (95% CI –2.28 to 0.51) for sotagliflozin 400 mg, and −0.56 (95% CI –1.95 to 0.84) for sotagliflozin 200 mg. Positive but nonsignificant effects were observed for empagliflozin (0.90, 95% CI –0.42 to 2.22), semaglutide (1.17, 95% CI –0.16 to 2.50), and canagliflozin 100 mg (1.71, 95% CI –0.31 to 3.72).

### 3.6 Change in UACR

Six studies reported the outcomes of changes in UACR.^27^, ^29^, ^30^, ^31^, ^34^, ^40^ After the analysis, two disconnected subnetworks were identified (Figure S5). One subnetwork contained only one DDP‐4 inhibitor (linagliptin), and another contained three SGLT‐2 inhibitors. Therefore, we performed a secondary analysis focusing on SGLT2 inhibitors (canagliflozin 300 mg, canagliflozin 100 mg, and dapagliflozin 10 mg). The forest plot (Figure S6) showed that canagliflozin 300 mg (ratio of means 0.57, 95% CI 0.42–0.78) and canagliflozin 100 mg (0.59, 95% CI 0.48–0.73) significantly reduced UACR compared with placebo, whereas dapagliflozin 10 mg (0.80, 95% CI 0.61–1.05) was not statistically significant.

### 3.7 Funnel plots and Egger's test

To assess potential publication bias, funnel plots were generated for all three outcomes (Figures S7–S9). For composite renal outcomes and change in eGFR, Egger's regression tests did not indicate small‐study effects (*p* > 0.05). For UACR, only seven studies were available; therefore, Egger's regression test was not performed due to limited statistical power. The funnel plot for UACR appeared symmetrical.

## 4 DISCUSSION

### 4.1 Key findings

This systematic review and network meta‐analysis provides the most updated and comprehensive drug‐specific evaluation of the renal effects of novel antidiabetic agents in patients with T2DM and CKD. Unlike previous analyses that mainly reported class‐level effects,^14^, ^45^ our study revealed clinically relevant heterogeneity across individual agents. Dapagliflozin showed the largest reduction in composite renal outcomes, canagliflozin produced the most pronounced reduction in albuminuria, and both dulaglutide 1.5 mg and semaglutide demonstrated significant renal benefits, whereas exenatide showed only a nonsignificant trend. DPP‐4 inhibitors provided no renal protection. These findings highlight that renal benefits are not uniform within drug classes and underscore the importance of drug‐level evaluation.

### 4.2 Comparison with previous studies

Our findings are consistent with large‐scale outcome trials confirming the renoprotective effects of SGLT2 inhibitors, including DAPA‐CKD,^11^ CREDENCE,^10^ and EMPA‐KIDNEY.^12^ These studies demonstrated significant reductions in kidney failure, albuminuria progression, and cardiovascular mortality, supporting the robust efficacy of this drug class. Earlier network meta‐analyses, such as those by Cao et al.^45^ and Yang et al.,^14^ also confirmed class‐wide benefits of SGLT2 inhibitors, but did not incorporate more recent evidence.

For GLP‐1 receptor agonists, our analysis aligns with evidence from the REWIND trial,^46^ where dulaglutide reduced albuminuria progression and improved renal outcomes. More recently, the FLOW trial demonstrated significant renal benefits of semaglutide, reinforcing the role of GLP‐1 receptor agonists in kidney protection.^43^ However, our results also highlight heterogeneity within this class: dulaglutide and semaglutide showed significant benefits, whereas exenatide exhibited only a nonsignificant trend.

By focusing on individual agents rather than drug classes, our study extends previous findings and identifies meaningful drug‐level differences, such as the stronger albuminuria reduction observed with canagliflozin compared with dapagliflozin, and the differential renal efficacy among GLP‐1 receptor agonists.

### 4.3 Potential mechanisms of renoprotection

The renoprotective effects of SGLT2 inhibitors are likely mediated by mechanisms beyond glucose lowering. These include restoration of tubuloglomerular feedback, reduction of intraglomerular pressure, improved renal oxygenation, and attenuation of oxidative stress.^49^, ^50^ Additional systemic effects, such as natriuresis, lower blood pressure, and weight reduction, also contribute to kidney protection.^47^, ^48^, ^49^, ^50^, ^51^ GLP‐1 receptor agonists may act through complementary pathways, including anti‐inflammatory and antifibrotic effects, improvement in blood pressure and body weight, and possible direct renal protection observed in preclinical studies.^6^, ^51^, ^52^, ^53^ The variable efficacy across individual GLP‐1 receptor agonists observed in our analysis may reflect differences in receptor affinity, pharmacokinetics, or trial populations. In contrast, the absence of benefit from DPP‐4 inhibitors is consistent with their lack of significant hemodynamic or renal‐specific effects.^4^, ^5^

### 4.4 Clinical implications

Our findings have direct clinical relevance for the management of patients with T2DM and CKD. SGLT2 inhibitors should remain the first‐line therapy given their consistent and robust renal benefits across trials.^7^, ^8^, ^9^, ^10^, ^11^, ^12^ Notably, dapagliflozin showed the greatest effect on composite renal outcomes, whereas canagliflozin demonstrated stronger reductions in albuminuria, suggesting that patient characteristics, such as baseline albuminuria, may guide optimal drug selection within this class. Among GLP‐1 receptor agonists, both dulaglutide 1.5 mg and semaglutide were associated with significant renal benefits, whereas exenatide showed only a nonsignificant trend, emphasizing that not all agents within this class are equivalent. These findings highlight the importance of individualized therapy and suggest that treatment decisions should consider not only glycemic control and cardiovascular outcomes but also the specific renal effects of individual drugs. In contrast, DPP‐4 inhibitors conferred no renal protection and therefore should not be prioritized for kidney outcomes in this population.

### 4.5 Limitations

This study has several limitations. First, most treatment comparisons relied on indirect evidence, as few head‐to‐head RCTs were available, which lowers the certainty of some estimates. Second, although the risk of bias was generally low to moderate, common concerns included selective reporting, incomplete outcome data, and outcome measurement. The certainty of evidence also varied: placebo‐controlled comparisons, particularly for dapagliflozin, canagliflozin, and semaglutide, were often rated high, whereas indirect and head‐to‐head comparisons were frequently downgraded to low or very low due to imprecision or inconsistency. Publication bias could not be fully excluded; although funnel plots and Egger's tests did not suggest small‐study effects for composite renal outcomes and eGFR, the limited number of studies for UACR reduced statistical power. Third, heterogeneity in study design, patient populations, and outcome definitions may have influenced the pooled results. Fourth, the analysis was based on study‐level aggregate data, preventing stratified analyses by baseline renal function or albuminuria. Fifth, the median follow‐up duration was relatively short, potentially underestimating long‐term renal effects. In addition, we excluded post‐hoc analyses, such as the SURPASS‐4 renal outcome study,^54^ because of their exploratory design and higher risk of bias. Finally, newer agents, including tirzepatide, were not included; future large‐scale, head‐to‐head RCTs are needed to define their comparative renal efficacy.

In conclusion, this network meta‐analysis reinforces SGLT2 inhibitors as the most reliable therapy for renal protection in patients with T2DM and CKD, while GLP‐1 receptor agonists provide additional but variable benefits, and DPP‐4 inhibitors show no effect. By evaluating agents at the drug level, our study highlights clinically meaningful heterogeneity and underscores the need for further head‐to‐head trials to refine treatment strategies and assess newer therapies such as tirzepatide.

## AUTHOR CONTRIBUTIONS

Design: Lin, Wu, and Chien contributed to the design and conceptualization of the study. Conduct/Data Collection: All authors coordinated data collection, interpretation, entry, and quality assurance. Wu and Chien also provided administrative, technical, and material support. Analysis: Lin and Shih performed the statistical analyses. Writing Manuscript: Lin, Hsu, and Shih drafted the manuscript. Wu and Chien critically reviewed the manuscript. All authors have read and approved the final version. Supervision was provided by Wu. Funding was obtained by Lin and Wu.

## FUNDING INFORMATION

This study was supported by research grants to Dr. Rong Lin from the Far Eastern Memorial Hospital, New Taipei City, Taiwan (FEMH‐2025‐C‐019, FEMH‐2023‐C‐010, FEMH‐2022‐C‐014), as well as by research grants to Dr. Hon‐Yen Wu from the National Health Research Institutes, Taiwan (NHRI‐EX112‐11026PI, NHRI‐EX114‐11433PI).

## CONFLICT OF INTEREST STATEMENT

The authors declare that there are no conflicts of interest related to this manuscript.

## Supporting information


**Appendix S1.** Study Protocol and Search Strategies.
**Appendix S2.** Estimations and Imputations for Missing Data References. References for Appendix 1 and Appendix 2.

**FIGURE S1.** PRISMA flow diagram of study selection.
**FIGURE S2.** Risk of bias assessment for included RCTs (Cochrane RoB 2 domains).
Green symbols represent a low risk of bias, yellow symbols represent some concerns of bias, and red symbols represent a high risk of bias. The figure was generated using robvis software to create risk‐of‐bias plots.
**FIGURE S3.** Three subnetworks of eGFR results.
**FIGURE S4.** Forest plot of the network meta‐analysis comparing interventions with placebo for the changes in eGFR.
**FIGURE S5.** Two subnetworks of UACR results.
**FIGURE S6.** Forest plot of the network meta‐analysis comparing interventions with placebo for the changes in UACR.
**FIGURE S7.** Funnel plot assessing publication bias for composite renal outcome.
**FIGURE S8.** Funnel plot assessing publication bias for eGFR outcomes.
**FIGURE S9.** Funnel plot assessing publication bias for UACR outcomes.

**TABLE S1.** PRISMA NMA checklist of items to include when reporting a systematic review involving a network meta‐analysis.
**TABLE S2.** Characteristics of the 20 included randomized controlled trials.
**TABLE S3.** GRADE evidence profile for Composite renal outcome in patients with T2DM and CKD.
**TABLE S4.** GRADE evidence profile for eGFR outcomes in patients with T2DM and CKD.
**TABLE S5.** GRADE evidence profile for UACR outcomes in patients with T2DM and CKD.
**TABLE S6.** P score of renal composite outcome.

## Data Availability

This study was based on data extracted from previously published randomized controlled trials and trial registries. PubMed, EMBASE, the Cochrane Central Register of Controlled Trials (CENTRAL), and ClinicalTrials.gov were systematically searched from inception to July 2025. All data analyzed are included in this article and its supplementary materials.
