# Supplementary material for: Comparison of the renal outcomes of novel antidiabetic agents in patients with type 2 diabetes with chronic kidney disease: A systematic review and network meta‐analysis of randomized controlled trials
Source: Diabetes Obes Metab. 2025 Oct 28;28(1):518–28. doi: 10.1111/dom.70224 (PMC12673457; doi:10.1111/dom.70224)
Supplement: Supplementary file 1 — Appendix S1. Study Protocol and Search Strategies. Appendix S2. Estimations and Imputations for Missing Data References. References for Appendix 1 and Appendix 2. [file DOM-28-518-s002.docx]

**Supplementary Material**

**Appendix 1.** Study Protocol and Search Strategies

**Appendix 2.** Estimations and Imputations for Missing Data

**References.** References for Appendix 1 and Appendix 2

**Appendix 1. Study Protocol and Search Strategies**

Study protocol for systematic review and network meta-analysis to assess the individual effects of DDP-4 inhibitors, GLP-1 agonists, and SGLT-2 inhibitors on kidney disease progression in type 2 diabetic patients with chronic kidney disease

**Objective**

To synthesize the results of all available randomized clinical trials that compare the individual effects of DDP-4 inhibitors, GLP-1 agonists, and SGLT-2 inhibitors versus placebo or usual treatment on kidney disease progression in type 2 diabetic patients with chronic kidney disease (CKD).

**Inclusion Criteria**

Study type

- Randomized control trial, parallel design, but not cross-over or cohort design
- Studies compared two treatment arms, any type of DDP-4 inhibitor or GLP-1 agonist or SGLT-2 inhibitor versus placebo (or usual treatment) to assess kidney disease outcomes in type 2 diabetes patients with CKD
- Study duration at least 24 weeks

Participants

- Eligible studies should have included type 2 diabetic adult participants older than 18 years
- We only included participants with type 2 diabetes, other types of diabetes will be excluded including type 1 diabetes mellitus, gestational diabetes, etc.
- Studies with any stage of CKD, except the dialysis participants, will be included
- Studies reporting outcomes from diabetic CKD subgroups will be included

Outcome measures

- Eligible studies should have reported at least one of the following outcomes:

1. composite renal outcome of 30% or greater reduction in eGFR
2. change of eGFR (mL/min/1.73 m2)
3. 50% or greater increase in serum creatinine level
4. percent changes of UAE or UACR from baseline to final levels
5. AKI, end-stage kidney disease or renal death

- The composite renal outcome of the 50% or greater increase in serum creatinine level, 30% reduction in GFR, AKI, ESRD or renal death will also be analyzed
- We will analyze any of the predefined primary and secondary outcomes
- We will only analyze outcomes reported during the in-trial follow-up period, not post hoc analysis
- ESRD is defined as the need for dialysis therapy or kidney transplantation.

Publication type

- Full-length articles in peer-reviewed journals will be eligible.

We will not restrict the language of publication

**Data extraction and quality assessment**

Two investigators (Rong Lin, Chia-Li Hsu) will independently extract the following information: details of study design, location and published year of study, patients’ characteristics (age, sex, BMI, mean of HbA1c), baseline renal function, target and achieved level of eGFR, UACR and composite renal outcome (ESRD, renal death, eGFR decline, >1.5 Creatinine increased) in each treatment arm, follow-up duration. The methodological quality of eligible trials will be evaluated independently by two investigators (Rong Lin, Chia-Li Hsu) using the website “ Robvis “ to create risk-of-bias plots for risk of bias (randomization, deviation from intended intervention, missing outcome data, measurement of outcome, selection of reported result)^1^. If relevant information about the design or outcomes is unclear, or if doubt exists about duplicate publications, the original authors will be contacted for clarification. Any disagreements between the two authors will be resolved through discussion.

**Data Synthesis and Analysis**

All data from each eligible study will be extracted by Covidence systematic review software^2^ and entered into a spreadsheet (Excel 2021; Microsoft Corporation, Redmond, WA).

**Search Strategies**

We will search the following electronic databases:

1. PubMed

2. Embase

3. Cochrane Library

There will be no restriction on language of publication. We will search additional studies in the reference lists of all identified publications, and only searching for RCT.

1. **PubMed:**

Search using the NCBI interface from the earliest available date of indexing through March 11, 2023 ( sodium-glucose transporter 2 inhibitors'/exp OR 'sodium-glucose transporter 2 inhibitors' OR 'tofogliflozin'/exp OR tofogliflozin OR 'apleway'/exp OR apleway OR 'deberza'/exp OR deberza OR 'empagliflozin'/exp OR empagliflozin OR 'jardiance'/exp OR jardiance OR 'dapagliflozin'/exp OR dapagliflozin OR 'farxiga'/exp OR farxiga OR 'forxiga'/exp OR forxiga OR 'canagliflozin'/exp OR canagliflozin OR 'invokana'/exp OR invokana OR 'sotagliflozin'/exp OR sotagliflozin OR 'luseogliflozin'/exp OR luseogliflozin OR 'lusefi'/exp OR lusefi OR 'ipragliflozin'/exp OR ipragliflozin OR 'suglat'/exp OR suglat OR 'remogliflozin'/exp OR remogliflozin OR 'sergliflozin'/exp OR sergliflozin OR 'ertugliflozin'/exp OR ertugliflozin OR 'dipeptidyl-peptidase iv inhibitors'/exp OR 'dipeptidyl-peptidase iv inhibitors' OR 'sitagliptin'/exp OR sitagliptin OR 'januvia'/exp OR januvia OR 'vildagliptin'/exp OR vildagliptin OR 'galvus'/exp OR galvus OR 'saxagliptin'/exp OR saxagliptin OR 'onglyza'/exp OR onglyza OR 'linagliptin'/exp OR linagliptin OR 'trajenta'/exp OR trajenta OR 'gemigliptin'/exp OR gemigliptin OR zemiglo OR 'anagliptin'/exp OR anagliptin OR 'suiny'/exp OR suiny OR 'teneligliptin'/exp OR teneligliptin OR 'tenelia'/exp OR tenelia OR 'alogliptin'/exp OR alogliptin OR 'nesina'/exp OR nesina OR 'vipidia'/exp OR vipidia OR 'trelagliptin'/exp OR trelagliptin OR zafatek OR 'omarigliptin'/exp OR omarigliptin OR 'evogliptin'/exp OR evogliptin OR suganon OR evodine OR 'gosogliptin'/exp OR gosogliptin OR 'dutogliptin'/exp OR dutogliptin OR 'glp-1 receptor agonists' OR 'glp-1-ra' OR 'incretin mimetics' OR 'glp-1 analogs' OR 'exenatide'/exp OR exenatide OR 'byetta'/exp OR byetta OR 'bydureon'/exp OR bydureon OR 'liraglutide'/exp OR liraglutide OR 'victoza'/exp OR victoza OR 'albiglutide'/exp OR albiglutide OR 'eperzan'/exp OR eperzan OR 'tanzeum'/exp OR tanzeum OR 'dulaglutide'/exp OR dulaglutide OR 'trulicity'/exp OR trulicity OR 'lixisenatide'/exp OR lixisenatide OR 'lyxumia'/exp OR lyxumia OR 'adlyxin'/exp OR adlyxin OR 'semaglutide'/exp OR semaglutide OR 'ozempic'/exp OR ozempic OR 'wegovy'/exp OR wegovy OR 'rybelsus'/exp OR rybelsus)

1. **Embase:**

Search using the Ovid interface from the earliest available date of indexing through March 11, 2023( 'sodium-glucose transporter 2 inhibitors'/exp OR 'sodium-glucose transporter 2 inhibitors' OR 'tofogliflozin'/exp OR tofogliflozin OR 'apleway'/exp OR apleway OR 'deberza'/exp OR deberza OR 'empagliflozin'/exp OR empagliflozin OR 'jardiance'/exp OR jardiance OR 'dapagliflozin'/exp OR dapagliflozin OR 'farxiga'/exp OR farxiga OR 'forxiga'/exp OR forxiga OR 'canagliflozin'/exp OR canagliflozin OR 'invokana'/exp OR invokana OR 'sotagliflozin'/exp OR sotagliflozin OR 'luseogliflozin'/exp OR luseogliflozin OR 'lusefi'/exp OR lusefi OR 'ipragliflozin'/exp OR ipragliflozin OR 'suglat'/exp OR suglat OR 'remogliflozin'/exp OR remogliflozin OR 'sergliflozin'/exp OR sergliflozin OR 'ertugliflozin'/exp OR ertugliflozin OR 'dipeptidyl-peptidase iv inhibitors'/exp OR 'dipeptidyl-peptidase iv inhibitors' OR 'sitagliptin'/exp OR sitagliptin OR 'januvia'/exp OR januvia OR 'vildagliptin'/exp OR vildagliptin OR 'galvus'/exp OR galvus OR 'saxagliptin'/exp OR saxagliptin OR 'onglyza'/exp OR onglyza OR 'linagliptin'/exp OR linagliptin OR 'trajenta'/exp OR trajenta OR 'gemigliptin'/exp OR gemigliptin OR zemiglo OR 'anagliptin'/exp OR anagliptin OR 'suiny'/exp OR suiny OR 'teneligliptin'/exp OR teneligliptin OR 'tenelia'/exp OR tenelia OR 'alogliptin'/exp OR alogliptin OR 'nesina'/exp OR nesina OR 'vipidia'/exp OR vipidia OR 'trelagliptin'/exp OR trelagliptin OR zafatek OR 'omarigliptin'/exp OR omarigliptin OR 'evogliptin'/exp OR evogliptin OR suganon OR evodine OR 'gosogliptin'/exp OR gosogliptin OR 'dutogliptin'/exp OR dutogliptin OR 'glp-1 receptor agonists' OR 'glp-1-ra' OR 'incretin mimetics' OR 'glp-1 analogs' OR 'exenatide'/exp OR exenatide OR 'byetta'/exp OR byetta OR 'bydureon'/exp OR bydureon OR 'liraglutide'/exp OR liraglutide OR 'victoza'/exp OR victoza OR 'albiglutide'/exp OR albiglutide OR 'eperzan'/exp OR eperzan OR 'tanzeum'/exp OR tanzeum OR 'dulaglutide'/exp OR dulaglutide OR 'trulicity'/exp OR trulicity OR 'lixisenatide'/exp OR lixisenatide OR 'lyxumia'/exp OR lyxumia OR 'adlyxin'/exp OR adlyxin OR 'semaglutide'/exp OR semaglutide OR 'ozempic'/exp OR ozempic OR 'wegovy'/exp OR wegovy OR 'rybelsus'/exp OR rybelsus)

1. **Cochrane Library:**

Searched using the Wiley interface from the earliest available date of indexing through March 11, 2023 ((sodium-glucose transporter 2 inhibitors OR tofogliflozin OR apleway OR deberza OR empagliflozi OR jardiance OR dapagliflozin OR forxiga OR canagliflozin or Invokana OR sotagliflozin OR luseogliflozin OR lusefi OR ipragliflozin OR suglat OR remogliflozin OR sergliflozin OR ertugliflozin OR dipeptidyl-peptidase iv inhibitors OR sitagliptin OR januvia OR vildagliptin OR galvus OR saxagliptin OR onglyza OR linagliptin OR trajenta OR gemigliptin OR zemiglo OR anagliptin OR suiny OR teneligliptin OR tenelia OR alogliptin OR nesina OR vipidia OR trelagliptin OR zafatek OR omarigliptin OR evogliptin OR suganon OR evodine OR gosogliptin OR dutogliptin OR glp-1 receptor agonists OR glp-1 analogs OR exenatide OR byetta OR bydureon OR liraglutide OR victoza OR albiglutide OR eperzan OR tanzeum OR dulaglutide OR trulicity OR lixisenatide OR lyxumia OR adlyxin OR semaglutid OR ozempic OR wegovy OR rybelsus)

(“renal insufficiency, chronic” [MeSH] or CKD or “chronic kidney disease” or “kidney disease” or “kidney failure” or CKF or “chronic kidney failure” or “renal failure” or CRF or CRD or “chronic renal disease” or “albuminuria” OR “urine albumin” OR “proteinuria” OR “urine protein”)

and type 2 diabetes and renal insufficiency)

**Appendix 2. Estimations and Imputations for Missing Data**

For essential data to pool the outcome of change of change in eGFR, we used estimation and imputation methods to reconstruct the missing values as recommended in the Cochrane Handbook ^3^. The formula: [standard deviation (SD) = √N × standard error (SE)] was used to obtain SD from SE, where N = sample size. The formula: [SD = √N × (upper limit of confidence interval - lower limit of confidence interval) / 3.92] was used to obtain SD from confidence intervals. According to the study reported by Hozo in 2005 ^4^, we obtain SD from interquartile range (IQR) using the formula:( Q3- Q1)/4 ,where Q1= quartile 1, Q3= quartile 3 as a way to obtain SD in study reported by Takashima in 2018 . In the study reported by Ruggenenti in 2005,10 we substituted the mean value from the median value, imputed the SD from the interquartile range (IQR) using the formula: [IQR = 1.35 × SD], and multiplied the monthly data by 12 to estimate the annual data. We used between-group P value to impute the SD for the study reported by Hayashi in 2010.11 For the SD of each study arm in the study reported by Schrier in 2014,12 we imputed it from confidence interval of the between-group mean difference, under the assumption that SD of each study arm was the same

**References:**

1. McGuinness, LA, Higgins, JPT. Risk-of-bias VISualization (robvis): An R package and Shiny web app for visualizing risk-of-bias assessments. Res Syn Meth. 2020; 1- 7. <https://doi.org/10.1002/jrsm.1411>
2. Covidence systematic review software, Veritas Health Innovation, Melbourne, Australia. Available at [www.covidence.org](http://www.covidence.org).
3. Higgins JP, Thomas J, Chandler J, Cumpston M, Li T, Page MJ, Welch VA, editor(s). Cochrane Handbook for Systematic Reviews of Interventions Version 6.2 (updated February 2021). Cochrane, 2021. Available from training.cochrane.org/handbook.
4. Hozo, S.P., Djulbegovic, B. & Hozo, I. Estimating the mean and variance from the median, range, and the size of a sample. *BMC Med Res Methodol* **5**, 13 (2005). https://doi.org/10.1186/1471-2288-5-13
