# Supplementary material for: Comparison of the renal outcomes of novel antidiabetic agents in patients with type 2 diabetes with chronic kidney disease: A systematic review and network meta‐analysis of randomized controlled trials
Source: Diabetes Obes Metab. 2025 Oct 28;28(1):518–28. doi: 10.1111/dom.70224 (PMC12673457; doi:10.1111/dom.70224)
Supplement: Supplementary file 2 — FIGURE S1. PRISMA flow diagram of study selection. FIGURE S2. Risk of bias assessment for included RCTs (Cochrane RoB 2 domains). Green symbols represent a low risk of bias, yellow symbols represent some concerns of bias, and red symbols represent a high risk of bias. The figure was generated using robvis software to create risk‐of‐bias plots. FIGURE S3. Three subnetworks of eGFR results. FIGURE S4. Forest plot of the network meta‐analysis comparing interventions with placebo for the changes in eGFR. FIGURE S5. Two subnetworks of UACR results. FIGURE S6. Forest plot of the network meta‐analysis comparing interventions with placebo for the changes in UACR. FIGURE S7. Funnel plot assessing publication bias for composite renal outcome. FIGURE S8. Funnel plot assessing publication bias for eGFR outcomes. FIGURE S9. Funnel plot assessing publication bias for UACR outcomes. [file DOM-28-518-s003.docx]

Supplementary figures

Figure S1. PRISMA flow diagram of study selection


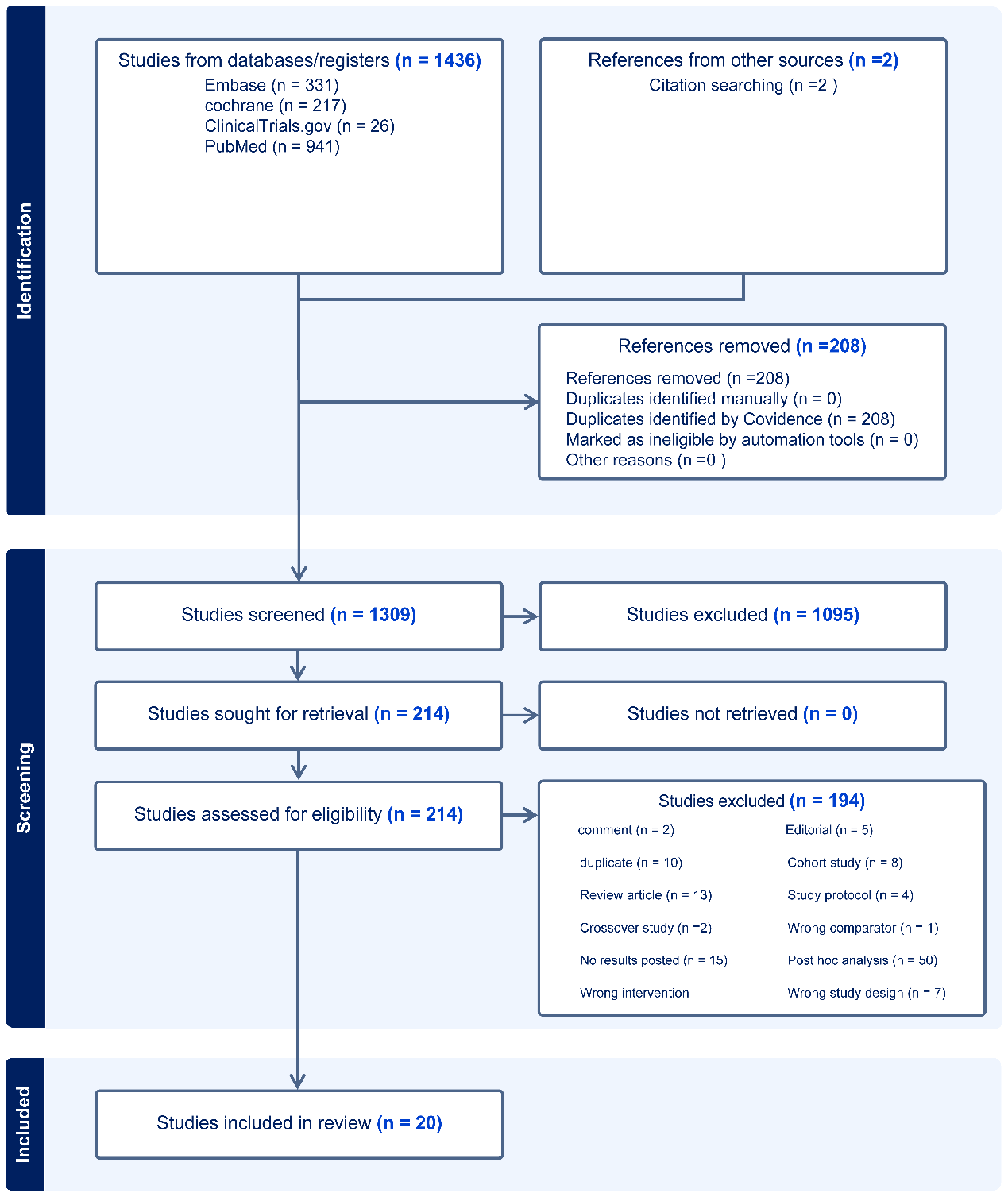


Figure S2. Risk of bias assessment for included RCTs (Cochrane RoB 2 domains).


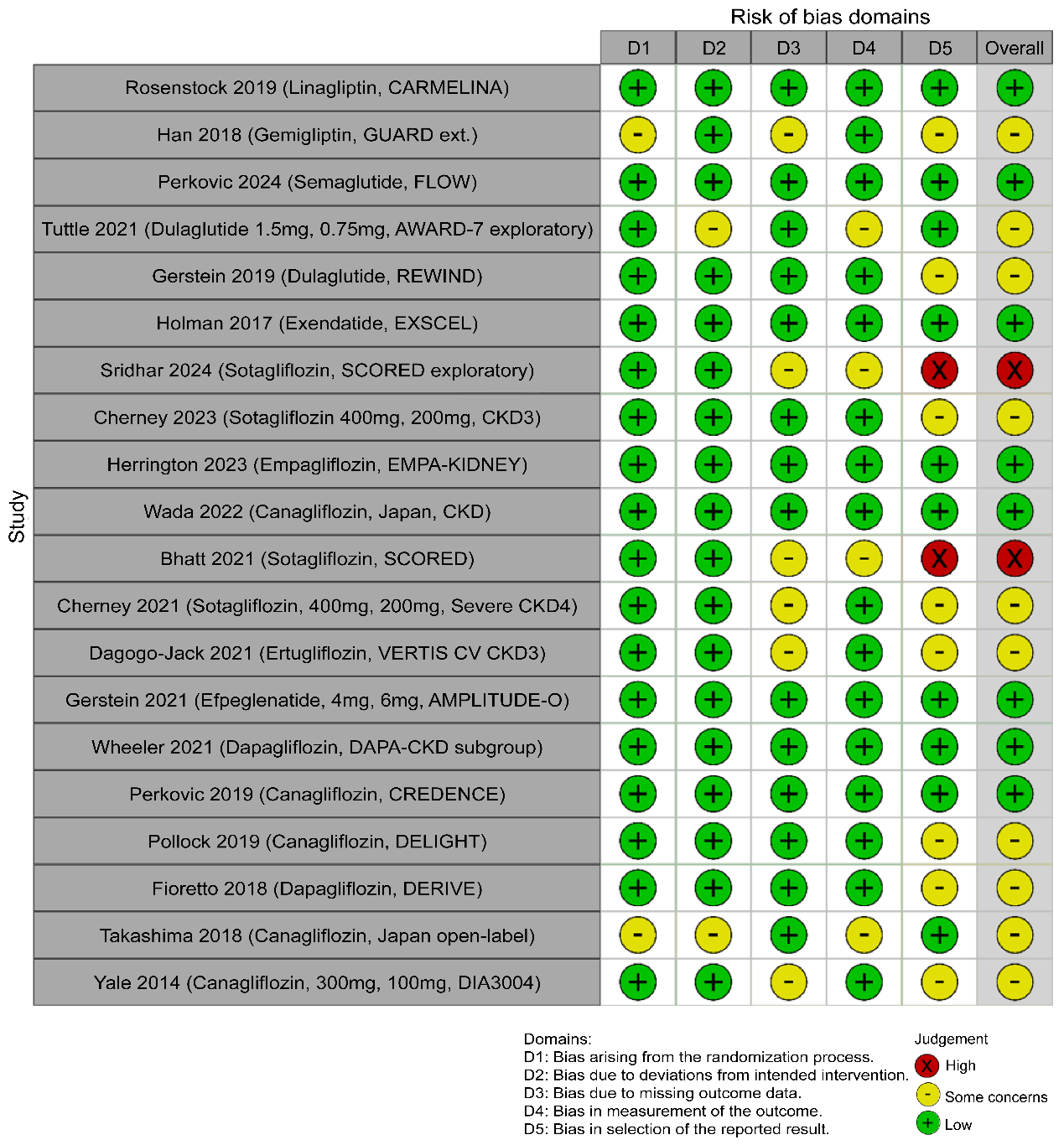


Green symbols represent a low risk of bias, yellow symbols represent some concerns of bias, and red symbols represent a high risk of bias. The figure was generated using robvis software to create risk-of-bias plots.

Figure S3. Three subnetworks of eGFR results


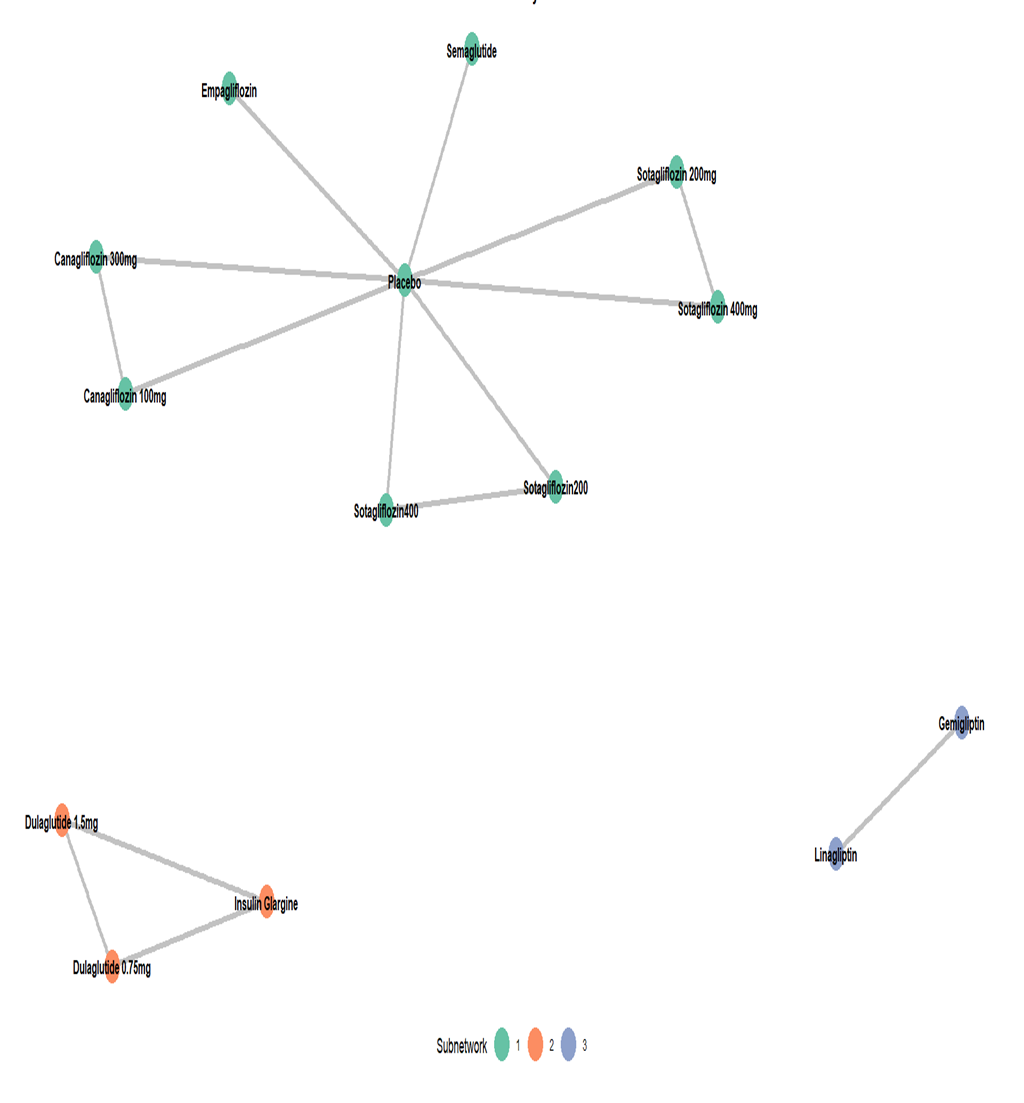


Figure S4. Forest plot of the network meta-analysis comparing interventions with placebo for the changes in eGFR.


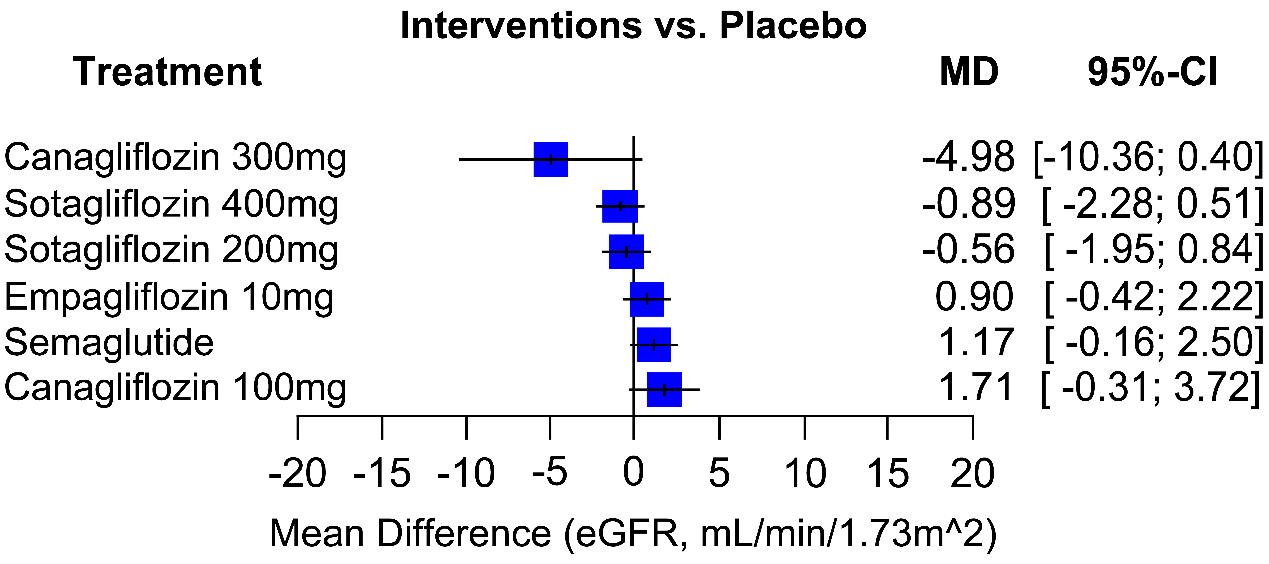


Figure S5. Two subnetworks of UACR results


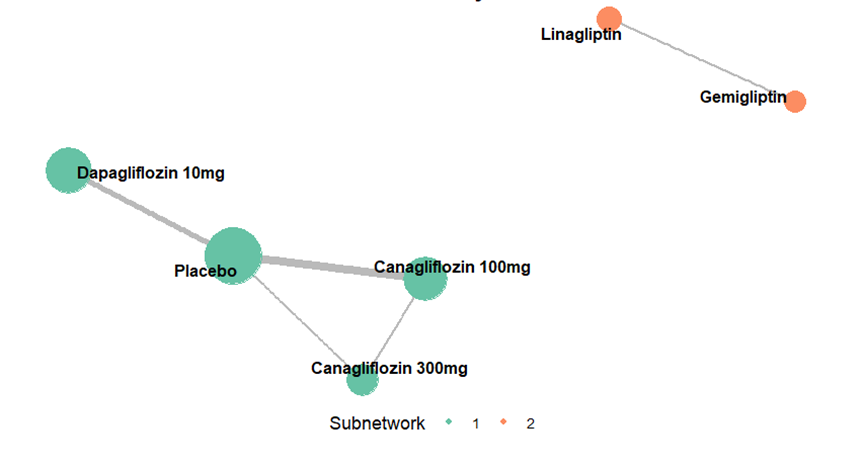


Figure S6. Forest plot of the network meta-analysis comparing interventions with placebo for the changes in UACR


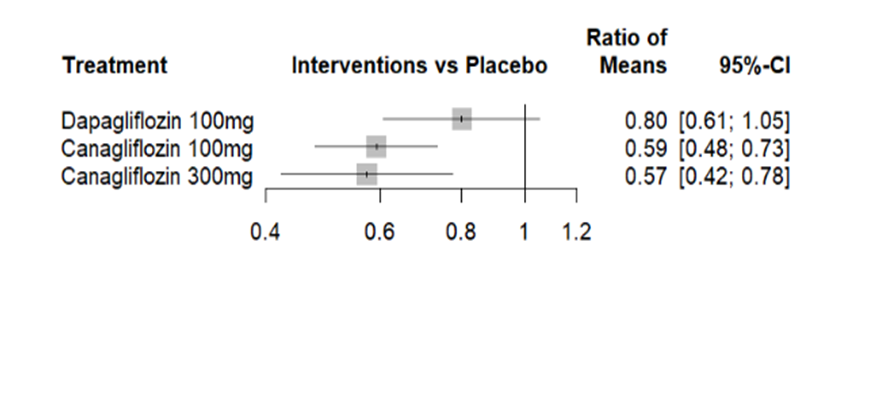


Figure S7. Funnel plot assessing publication bias for composite renal outcome


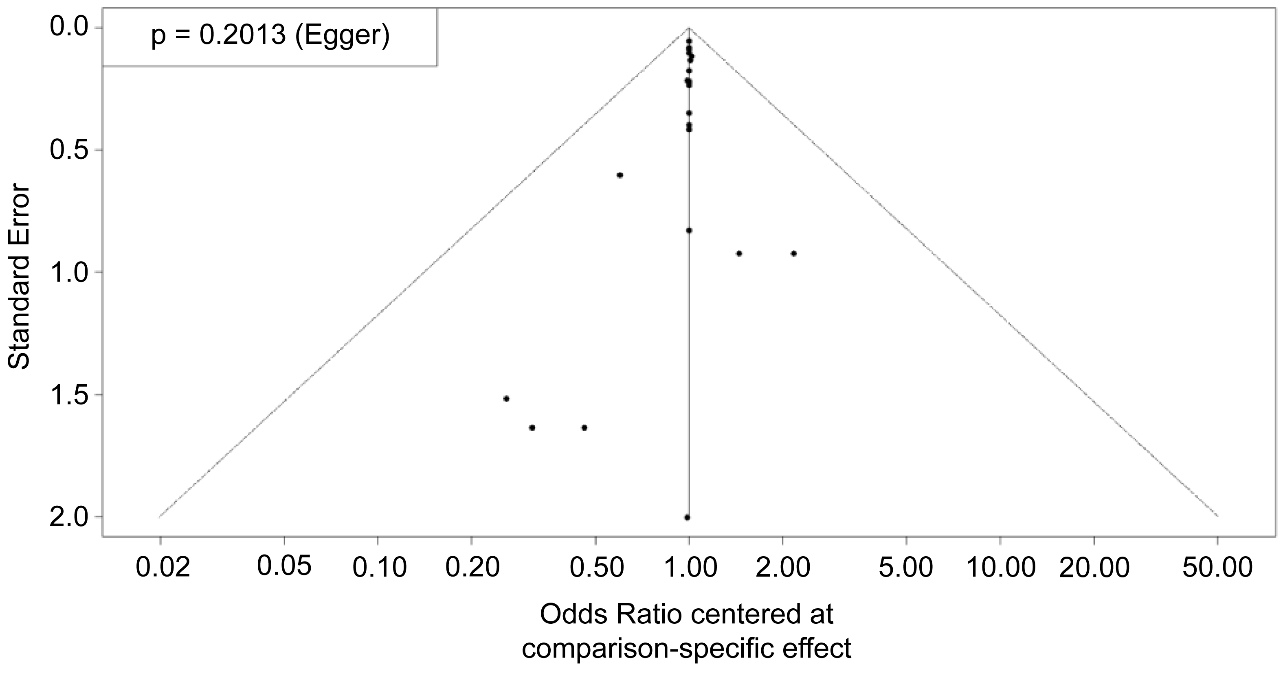


Each dot represents an individual RCT. The x-axis indicates the odds ratio (log scale) for composite renal outcomes, the y-axis represents the standard error. The funnel plot appeared symmetrical, and Egger’s regression test did not indicate small-study effects (p > 0.05).

Figure S8 Funnel plot assessing publication bias for eGFR outcomes


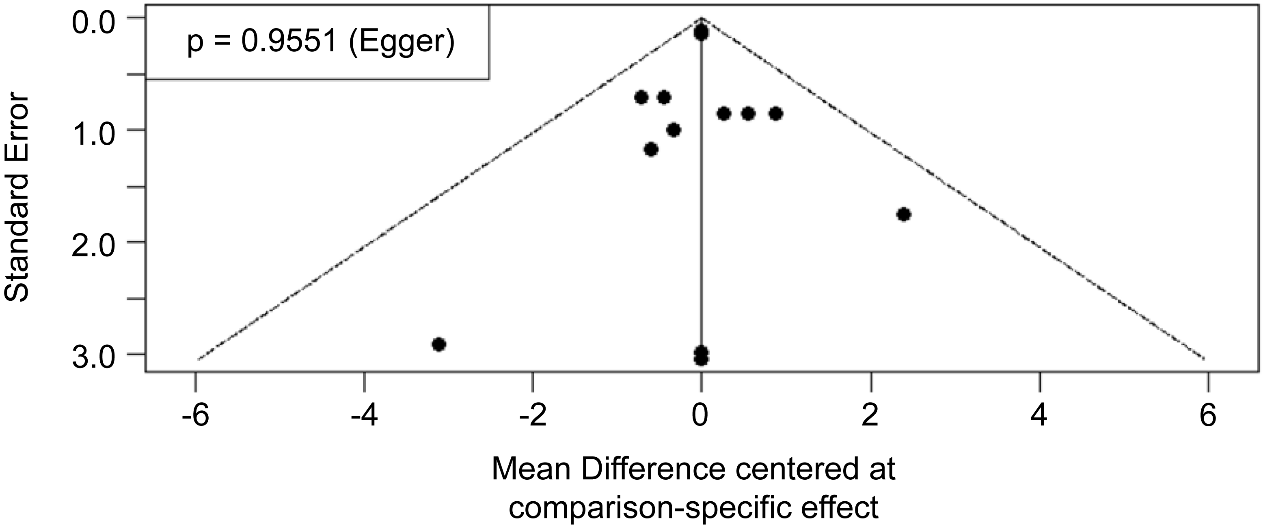


Each dot represents an individual RCT. The x-axis indicates the mean difference (MD) in eGFR (mL/min/1.73 m²), and the y-axis represents the standard error. The funnel plot appeared symmetrical and Egger’s regression test did not show evidence of small-study effects (p > 0.05).

Figure S9. Funnel plot assessing publication bias for UACR outcomes
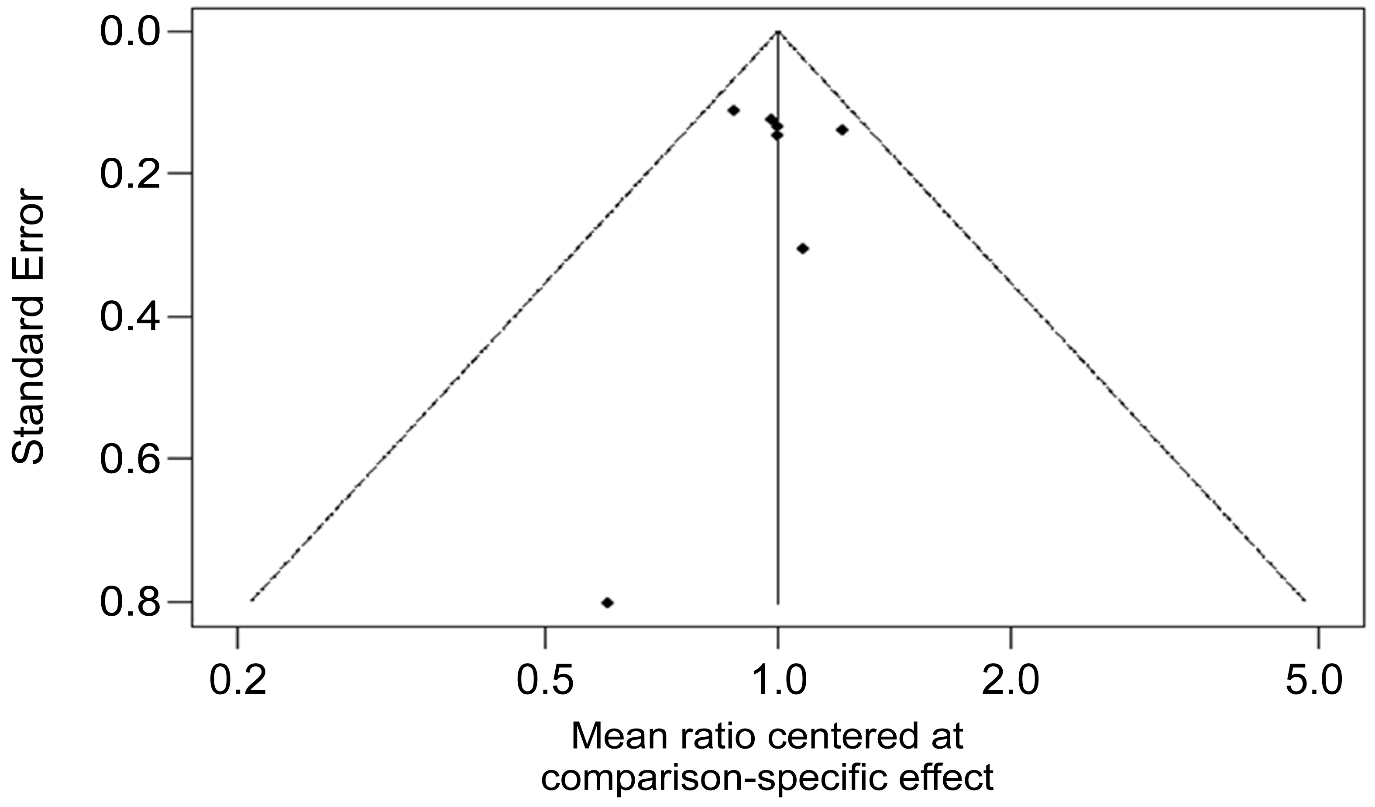


Each dot represents an individual RCT. The x-axis indicates the ratio of fold change in UACR, and the y-axis represents the standard error. Egger’s regression test was not performed because only seven studies were available. The funnel plot appeared symmetrical without obvious asymmetry.
