# Supplementary material for: Comparison of the renal outcomes of novel antidiabetic agents in patients with type 2 diabetes with chronic kidney disease: A systematic review and network meta‐analysis of randomized controlled trials
Source: Diabetes Obes Metab. 2025 Oct 28;28(1):518–28. doi: 10.1111/dom.70224 (PMC12673457; doi:10.1111/dom.70224)
Supplement: Supplementary file 3 — TABLE S1. PRISMA NMA checklist of items to include when reporting a systematic review involving a network meta‐analysis. TABLE S2. Characteristics of the 20 included randomized controlled trials. TABLE S3. GRADE evidence profile for Composite renal outcome in patients with T2DM and CKD. TABLE S4. GRADE evidence profile for eGFR outcomes in patients with T2DM and CKD. TABLE S5. GRADE evidence profile for UACR outcomes in patients with T2DM and CKD. TABLE S6. P score of renal composite outcome. [file DOM-28-518-s001.docx]

**Table S1: PRISMA NMA Checklist of Items to Include When Reporting A Systematic Review Involving a Network Meta-analysis:**

(

| **Section/Topic** | **Item #** | **Checklist Item** | **Reported on Page #** |
| --- | --- | --- | --- |
| **TITLE** |  |  |  |
| Title | 1 | Identify the report as a systematic review incorporating a network meta-analysis (or related form of meta-analysis). | **1** |
| **ABSTRACT** |  |  |  |
| Structured summary | 2 | Provide a structured summary including, as applicable:  **Background:** main objectives  **Methods:** data sources; study eligibility criteria, participants, and interventions; study appraisal; and synthesis methods, such as network meta-analysis.  **Results:** number of studies and participants identified; summary estimates with corresponding confidence/credible intervals; treatment rankings may also be discussed. Authors may choose to summarize pairwise comparisons against a chosen treatment included in their analyses for brevity.  **Discussion/Conclusions:** limitations; conclusions and implications of findings.  **Other:** primary source of funding; systematic review registration number with registry name. | 3-4 |
| **INTRODUCTION** |  |  |  |
| Rationale | 3 | Describe the rationale for the review in the context of what is already known, including mention of why a network meta-analysis has been conducted. | 4 |
| Objectives | 4 | Provide an explicit statement of questions being addressed, with reference to participants, interventions, comparisons, outcomes, and study design (PICOS). | 5-6 |
| **METHODS** |  |  |  |
| Protocol and registration | 5 | Indicate whether a review protocol exists and if and where it can be accessed (e.g., Web address); and, if available, provide registration information, including registration number. | 5 |
| Eligibility criteria | 6 | Specify study characteristics (e.g., PICOS, length of follow-up) and report characteristics (e.g., years considered, language, publication status) used as criteria for eligibility, giving rationale. Clearly describe eligible treatments included in the treatment network, and note whether any have been clustered or merged into the same node (with justification). | 5-6 |
| Information sources | 7 | Describe all information sources (e.g., databases with dates of coverage, contact with study authors to identify additional studies) in the search and date last searched. | 5-6 |
| Search | 8 | Present full electronic search strategy for at least one database, including any limits used, such that it could be repeated. | 5-6 |
| Study selection | 9 | State the process for selecting studies (i.e., screening, eligibility, included in systematic review, and, if applicable, included in the meta-analysis). | 6 |
| Data collection process | 10 | Describe method of data extraction from reports (e.g., piloted forms, independently, in duplicate) and any processes for obtaining and confirming data from investigators. | 6 |
| Data items | 11 | List and define all variables for which data were sought (e.g., PICOS, funding sources) and any assumptions and simplifications made. | 6 |
| **Geometry of the network** | **S1** | Describe methods used to explore the geometry of the treatment network under study and potential biases related to it. This should include how the evidence base has been graphically summarized for presentation, and what characteristics were compiled and used to describe the evidence base to readers. | **6-7** |
| Risk of bias within individual studies | 12 | Describe methods used for assessing risk of bias of individual studies (including specification of whether this was done at the study or outcome level), and how this information is to be used in any data synthesis. | 6-7 |
| Summary measures | 13 | State the principal summary measures (e.g., risk ratio, difference in means). Also describe the use of additional summary measures assessed, such as treatment rankings and surface under the cumulative ranking curve (SUCRA) values, as well as modified approaches used to present summary findings from meta-analyses. | 8 |
| Planned methods of analysis | 14 | Describe the methods of handling data and combining results of studies for each network meta-analysis. This should include, but not be limited to:   - Handling of multi-arm trials; - Selection of variance structure; - Selection of prior distributions in Bayesian analyses; and - Assessment of model fit. | 7-8 |
| **Assessment of Inconsistency** | **S2** | Describe the statistical methods used to evaluate the agreement of direct and indirect evidence in the treatment network(s) studied. Describe efforts taken to address its presence when found. | 8 |
| Risk of bias across studies | 15 | Specify any assessment of risk of bias that may affect the cumulative evidence (e.g., publication bias, selective reporting within studies). | 6-7 |
| Additional analyses | 16 | Describe methods of additional analyses if done, indicating which were pre-specified. This may include, but not be limited to, the following:   - Sensitivity or subgroup analyses; - Meta-regression analyses; - Alternative formulations of the treatment network; and - Use of alternative prior distributions for Bayesian analyses (if applicable). | **No** |
| **RESULTS†** |  |  |  |
| Study selection | 17 | Give numbers of studies screened, assessed for eligibility, and included in the review, with reasons for exclusions at each stage, ideally with a flow diagram. | 8, figure S1 |
| **Presentation of network structure** | **S3** | Provide a network graph of the included studies to enable visualization of the geometry of the treatment network. | 36 |
| **Summary of network geometry** | **S4** | Provide a brief overview of characteristics of the treatment network. This may include commentary on the abundance of trials and randomized patients for the different interventions and pairwise comparisons in the network, gaps of evidence in the treatment network, and potential biases reflected by the network structure. | 26-32, table 1, table S2, |
| Study characteristics | 18 | For each study, present characteristics for which data were extracted (e.g., study size, PICOS, follow-up period) and provide the citations. | 8-9,table 1, table S2, |
| Risk of bias within studies | 19 | Present data on risk of bias of each study and, if available, any outcome level assessment. | 9, Figure S2 |
| Results of individual studies | 20 | For all outcomes considered (benefits or harms), present, for each study: 1) simple summary data for each intervention group, and 2) effect estimates and confidence intervals. Modified approaches may be needed to deal with information from larger networks. | Table S2 |
| Synthesis of results | 21 | Present results of each meta-analysis done, including confidence/credible intervals. In larger networks, authors may focus on comparisons versus a particular comparator (e.g. placebo or standard care), with full findings presented in an appendix. League tables and forest plots may be considered to summarize pairwise comparisons. If additional summary measures were explored (such as treatment rankings), these should also be presented. | Figure 2 (forest plot, page 37); table 2 (league table, page 34-35); ranking, table Figure S6 |
| **Exploration for inconsistency** | **S5** | Describe results from investigations of inconsistency. This may include such information as measures of model fit to compare consistency and inconsistency models, P values from statistical tests, or summary of inconsistency estimates from different parts of the treatment network. | 11,Tables S3–S5 |
| Risk of bias across studies | 22 | Present results of any assessment of risk of bias across studies for the evidence base being studied. | 9,10, Figure S2 |
| Results of additional analyses | 23 | Give results of additional analyses, if done (e.g., sensitivity or subgroup analyses, meta-regression analyses, alternative network geometries studied, alternative choice of prior distributions for Bayesian analyses, and so forth). | NA |
| **DISCUSSION** |  |  |  |
| Summary of evidence | 24 | Summarize the main findings, including the strength of evidence for each main outcome; consider their relevance to key groups (e.g., healthcare providers, users, and policy-makers). | 12-15 |
| Limitations | 25 | Discuss limitations at study and outcome level (e.g., risk of bias), and at review level (e.g., incomplete retrieval of identified research, reporting bias). Comment on the validity of the assumptions, such as transitivity and consistency. Comment on any concerns regarding network geometry (e.g., avoidance of certain comparisons). | 15 |
| Conclusions | 26 | Provide a general interpretation of the results in the context of other evidence, and implications for future research. | 15-16 |
| **FUNDING** |  |  |  |
| Funding | 27 | Describe sources of funding for the systematic review and other support (e.g., supply of data); role of funders for the systematic review. This should also include information regarding whether funding has been received from manufacturers of treatments in the network and/or whether some of the authors are content experts with professional conflicts of interest that could affect use of treatments in the network. | 16 |

PICOS = population, intervention, comparators, outcomes, study design.

* Text in italics indicateS wording specific to reporting of network meta-analyses that has been added to guidance from the PRISMA statement.

† Authors may wish to plan for use of appendices to present all relevant information in full detail for items in this section.

Table S2 Characteristics of the 20 included randomized controlled trials

| **Author, Year (Study)** | **Drug / Comparator** | **Sample size (N)** | **Population (T2DM + CKD stage)** | **Follow-up (weeks)** | **Key Renal Outcomes** | **Main Findings** |
| --- | --- | --- | --- | --- | --- | --- |
| **DPP-4 inhibitor trials** | | | | | | |
| Rosenstock 2019 (CARMELINA)) | Linagliptin vs Placebo (CARMELINA trial subgroup) | ~6979 overall (subset with CKD: eGFR <60 or albuminuria) | T2DM with high CV risk and CKD (mean eGFR 55 mL/min/1.73 m²) | Median 2.2 years | Composite renal outcome (renal death, ESRD, sustained eGFR decline ≥40%), UACR | Linagliptin did not reduce risk of composite renal outcome compared with placebo; slowed albuminuria progression in some subgroups; overall renal effects neutral; safe in advanced CKD |
| Han 2018 (GUARD extension) | Gemigliptin 50 mg vs Linagliptin 5 mg (after placebo) | 100 randomized (48 vs 52 in extension) | T2DM with moderate-to-severe renal impairment (eGFR 15–59 mL/min/1.73 m²) | 40-week extension (total 52 weeks) | eGFR, UACR, albuminuria | HbA1c reduced similarly in both groups (−1.00% vs −0.65%); eGFR declined modestly in both groups (−3.9 vs −1.9 mL/min/1.73 m²); UACR changes were not significant; both drugs reduced urinary type IV collagen; safety profile and hypoglycemia risk were comparable |
| **GLP-1 receptor agonists** | | | | | | |
| Perkovic 2024 (FLOW) | Semaglutide 1.0 mg weekly vs Placebo | 3533 | T2DM + CKD | Median 3.4 years (early termination for efficacy) | Composite: kidney failure (dialysis, transplantation, eGFR <15 mL/min/1.73m²), ≥50% eGFR reduction from baseline, or death from kidney-related/cardiovascular causes | 24% risk reduction (HR 0.76, 95% CI 0.66-0.88, p=0.0003). Kidney-specific components HR 0.79 (0.66-0.94). CV death HR 0.71 (0.56-0.89). All-cause mortality HR 0.80 (0.67-0.95). Trial stopped early for efficacy. |
| Tuttle 2021 (AWARD-7 exploratory) | Dulaglutide 0.75 mg or 1.5 mg weekly vs Insulin glargine (basal) | 576 | T2DM + CKD stage 3–4 (eGFR 15–60); stratified by albuminuria (normo-, micro-, macroalbuminuria) | 52 | Composite of ≥40% eGFR decline, ESKD, or kidney disease death | Dulaglutide 1.5 mg weekly lowered risk of ≥40% eGFR decline/ESKD vs insulin glargine (HR 0.45). Benefit was most evident in macroalbuminuria (HR 0.25), with no kidney deaths; no significant effect in normo-/microalbuminuria. |
| Gerstein (2019) (REWIND) | Dulaglutide 1.5 mg vs Placebo | 9901 (4949 vs 4952) | T2DM + CV risk factors | Median 5.4 years (IQR 5.1-5.9) | Composite: new macroalbuminuria (UACR >33.9 mg/mmol), sustained ≥30% eGFR decline, or chronic renal replacement therapy | 5% risk reduction (HR 0.85, 95% CI 0.77-0.93, p=0.0004). Greatest effect on macroalbuminuria (HR 0.77, p<0.0001). Effect partly mediated by HbA1c and BP reduction. |
| Holman 2017 (EXSCEL) | Exenatide ER 2 mg weekly vs Placebo | 14,752 (wide CV risk; ~15% eGFR <60) | T2DM with or without established CVD, some CKD | Median 3.2 years (IQR 2.2-4.4) | Renal secondary endpoints (eGFR decline, albuminuria progression, ESRD) | Exenatide was noninferior for CV outcomes; renal outcomes neutral overall, no significant slowing of CKD progression. |
|  |  |  |  |  |  |  |
| SGLT-2 inhibitors | | | | | | |
| Sridhar 2024 (SCORED exploratory analysis) | Sotagliflozin 200–400 mg vs Placebo | 10,584 | T2DM with CKD (eGFR 25–60, wide UACR range), CV risk | Median 16 months | Kidney composite (≥50% ↓eGFR, eGFR<15, dialysis, transplant); Cardiorenal composite (kidney composite + CV/kidney death) | Sotagliflozin ↓ kidney composite risk 38% (HR 0.62, p<0.001); ↓ cardiorenal composite risk 23% (HR 0.77, p=0.002). Benefit consistent across eGFR/HbA1c, strongest in high albuminuria. No ↑ in AKI. |
| Cherney 2023 | Sotagliflozin 200 & 400 mg vs Placebo | 787 | T2DM with CKD stage 3 (eGFR 30–59) | 52 weeks | UACR (≥A2 albuminuria subgroup), eGFR slope, renal events | At 26 wks, significant ↓UACR (–30% to –36%) with both doses, not sustained at 52 wks. HbA1c lowering only with 400 mg at 26 wks. eGFR dip then stable; no sustained ≥50% decline or ESRD observed. Safety similar to placebo, with ↑ genital infections, diarrhea, and volume depletion. |
| Herrington 2023 (EMPA-KIDNEY) | Empagliflozin 10 mg daily vs Placebo | 6609 | CKD with or without diabetes; eGFR 20–45, or eGFR 45–<90 with UACR ≥200 | Median 2.0 years | Primary: progression of CKD (ESKD, sustained ↓eGFR ≥40%, eGFR <10, renal death) or CV death | Empagliflozin reduced risk of kidney disease progression or CV death (HR 0.72, 95% CI 0.64–0.82, p<0.001). Benefits consistent in diabetic and non-diabetic CKD, across eGFR/albuminuria subgroups. eGFR slope analyses showed slowing of long-term decline. Safety profile comparable to placebo. |
| Wada 2022 | Canagliflozin 100 mg vs Placebo | 308 | Japanese T2DM + CKD (eGFR 30–<90, UACR ≥300 mg/g) | 104 weeks | ≥30% eGFR decline, UACR, ESRD events | Cana reduced ≥30% eGFR decline risk (18.2% vs 29.5%); UACR ↓39% |
| Bhatt 2021 (SCORED) | Sotagliflozin 200–400 mg QD vs Placebo | 10,584 | T2DM + CKD, eGFR 25–60 (CKD G3a–G4) | Median 70 weeks (16 mo) | Composite renal endpoint (≥50% sustained eGFR decline, ESKD, or renal death) | Trial stopped early for funding; sotagliflozin reduced total HF events; renal outcomes limited, interpretation constrained by early termination. |
| Cherney 2021 | Sotagliflozin 200 or 400 mg QD vs Placebo | 277 | T2DM + advanced CKD stage 4 (eGFR 15–30) | 52 | eGFR change; UACR (subgroup) | HbA1c lowering limited at stage 4 CKD; eGFR change not different vs placebo; UACR trend favorable; safety consistent with SCORED. |
| Dagogo-Jack 2021 | Sotagliflozin vs Placebo | ~1,000+ (phase 3 program) | T2DM + CKD subgroups (post-hoc pooled analysis) | 52 | eGFR, UACR, safety | Confirmed glycemic efficacy and renal safety across CKD subgroups; no new renal safety signals in CKD patients. |
| Gerstein 2021 (AMPLITUDE-O) | Efpeglenatide 4 or 6 mg weekly vs Placebo (GLP-1 RA) | 4,076 | T2DM + CVD or CKD (eGFR 25–60) | Median 94 weeks (1.8 years) | Composite renal endpoint (macroalbuminuria, ≥40% eGFR decline, ESKD, renal death) | Efpeglenatide reduced renal composite events by 32% (HR 0.68, 95% CI 0.57–0.79); suggests complementary reno-protective effect independent of SGLT-2 use. |
| Wheeler 2021 (DAPA-CKD subgroup) | Dapagliflozin 10 mg vs Placebo | 4,304 | CKD with and without T2DM; eGFR 25–75; UACR 200–5000 | Median 2.4 years (~125 weeks) | Primary composite (sustained ≥50% eGFR decline, ESKD, or renal/CV death) | Benefits of dapagliflozin consistent regardless of diabetes status; reduced major adverse kidney and CV events. |
| Perkovic 2019 (CREDENCE) | Canagliflozin 100 mg vs Placebo | 4401 | T2DM + CKD, eGFR 30–90, albuminuria >300 mg/g, on ACEi/ARB | Median 2.62 years | Composite (ESKD, doubling creatinine, renal/CV death) | Reduced renal composite by 30%; slowed eGFR decline; reduced albuminuria |
| Pollock 2019 (DELIGHT) | Dapagliflozin 10 mg vs Placebo | 461 | T2DM + CKD stage 3–4, eGFR 25–75, UACR 30–3500, on ACEi/ARB | 24 weeks | Change in UACR | Dapa ↓UACR 21%, Dapa+Saxa ↓UACR 38%; HbA1c reduced; safe |
| Fioretto 2018 (DERIVE) | Dapagliflozin 10 mg vs Placebo | 321 | T2DM + CKD stage 3A (eGFR 45–59) | 24 weeks | eGFR, UACR (exploratory) | Dapa improved HbA1c, BP, weight; renal effect modest, benefit in albuminuria subgroup |
| Takashima 2018 | Canagliflozin 100 mg vs Placebo | 40 | T2DM + early CKD (eGFR ~60–90) | 24 weeks | UACR change | Cana ↓UACR; small study, exploratory |
| Yale 2014 | Canagliflozin 100/300 mg vs Placebo | 269 | T2DM + CKD stage 3 | 52 weeks | UACR, eGFR | Cana 100/300 mg reduced albuminuria vs placebo |

Table S3. GRADE evidence profile for Composite renal outcome in patients with T2DM and CKD

| **Comparison** | **Network estimate** | | | **Final rating** | **Reasons for downgrading** |
| --- | --- | --- | --- | --- | --- |
|  | **Relative estimate** | | |  |  |
|  | **Point estimate** | **CI lower limit** | **CI upper limit** |  |  |
| Semaglutide v.s Placebo | 0.76 | 0.65 | 0.9 | HIGH⊕⊕⊕ |  |
| Sotagliflozin 200mg v.s Placebo | 0.8 | 0.21 | 3.03 | VERY LOW○○○ | RoB, Imprecisionx2 |
| Sotagliflozin 400mg v.s Placebo | 0.72 | 0.54 | 0.96 | MODERATE○⊕⊕ | RoB |
| Canagliflozin 100mg v.s Placebo | 0.64 | 0.51 | 0.8 | HIGH⊕⊕⊕ |  |
| Dapagliflozin 10mg v.s Placebo | 0.55 | 0.42 | 0.72 | HIGH⊕⊕⊕ |  |
| Dulaglutide 0.75mg v.s Placebo | 1.43 | 0.63 | 3.27 | VERY LOW○○○ | RoB,Indirectnessx2, Imprecisionx2, Intransitivityx2 |
| Dulaglutide 1.5mg v.s Placebo | 0.86 | 0.77 | 0.95 | MODERATE○⊕⊕ | Indirectness |
| Efpeglenatide 4 + 6mg v.s Placebo | 0.66 | 0.55 | 0.78 | HIGH⊕⊕⊕ |  |
| Empagliflozin v.s Placebo | 0.65 | 0.53 | 0.8 | MODERATE○⊕⊕ | Indirectness |
| Ertugliflozin 5mg v.s Placebo | 0.71 | 0.46 | 1.11 | HIGH⊕⊕⊕ |  |
| Ertugliflozin 15mg v.s Placebo | 0.89 | 0.57 | 1.36 | HIGH⊕⊕⊕ |  |
| Exenatide v.s Placebo | 0.86 | 0.61 | 1.22 | MODERATE○⊕⊕ | Indirectness |
| Insulin Glargine v.s Placebo | 1.89 | 0.86 | 4.16 | VERY LOW○○○ | RoB,Indirectnessx2, Imprecisionx2, Intransitivityx2 |
| Linagliptin v.s Placebo | 1.07 | 0.91 | 1.26 | HIGH⊕⊕⊕ |  |
| Canagliflozin 100mg v.s Semaglutide | 0.84 | 0.64 | 1.1 | HIGH⊕⊕⊕ |  |
| Dulaglutide 0.75mg v.s Semaglutide | 1.88 | 0.81 | 4.34 | VERY LOW○○○ | RoB,Indirectnessx2, Imprecisionx2, Intransitivityx2 |
| Efpeglenatide 4 + 6mg v.s Dulaglutide 1.5mg | 0.77 | 0.62 | 0.94 | VERY LOW○○○ | Indirectness, Intransitivityx2 |
| Exenatide v.s Dulaglutide 1.5mg | 1 | 0.69 | 1.43 | VERY LOW○○○ | Indirectness, Intransitivityx2 |
| Insulin Glargine v.s Dulaglutide 1.5mg | 2.21 | 1.01 | 4.83 | VERY LOW○○○ | RoB,Indirectnessx2, Imprecisionx2 |
| Sotagliflozin 400mg v.s Sotagliflozin 200mg | 0.89 | 0.24 | 3.36 | VERY LOW○○○ | RoB, Imprecisionx2 |
| Ertugliflozin 15mg v.s Ertugliflozin 5mg | 1.24 | 0.78 | 1.96 | HIGH⊕⊕⊕ |  |
| Semaglutide v.s Sotagliflozin 200mg | 0.95 | 0.25 | 3.61 | VERY LOW○○○ | RoB, Imprecisionx2 |
| Semaglutide v.s Sotagliflozin 400mg | 1.06 | 0.76 | 1.49 | LOW  ○○⊕ | RoB, Intransitivity |
| Semaglutide v.s Dapagliflozin 10mg | 1.38 | 1.02 | 1.88 | MODERATE○⊕⊕ | Intransitivity |
| Semaglutide v.s Dulaglutide 1.5mg | 0.89 | 0.73 | 1.08 | LOW  ○○⊕ | Indirectness, Intransitivity |
| Semaglutide v.s Efpeglenatide 4 + 6mg | 1.16 | 0.91 | 1.48 | MODERATE○⊕⊕ | Intransitivity |
| Semaglutide v.s Empagliflozin | 1.17 | 0.9 | 1.52 | LOW  ○○⊕ | Indirectness, Intransitivity |
| Semaglutide v.s Ertugliflozin 5mg | 1.07 | 0.67 | 1.71 | HIGH⊕⊕⊕ |  |
| Semaglutide v.s Ertugliflozin 15mg | 0.86 | 0.54 | 1.37 | HIGH⊕⊕⊕ |  |
| Semaglutide v.s Exenatide | 0.89 | 0.61 | 1.3 | LOW  ○○⊕ | Indirectness, Intransitivity |
| Semaglutide v.s Insulin Glargine | 0.4 | 0.18 | 0.9 | VERY LOW○○○ | RoB,Indirectnessx2, Imprecisionx2, Intransitivityx2 |
| Semaglutide v.s Linagliptin | 0.71 | 0.57 | 0.9 | HIGH⊕⊕⊕ |  |
| Sotagliflozin 200mg v.s Canagliflozin 100mg | 0.8 | 0.21 | 3.06 | VERY LOW○○○ | RoB, Imprecisionx2 |
| Sotagliflozin 200mg v.s Dapagliflozin 10mg | 0.69 | 0.18 | 2.65 | VERY LOW○○○ | RoB, Imprecisionx2 |
| Sotagliflozin 200mg v.s Dulaglutide 0.75mg | 1.78 | 0.37 | 8.49 | VERY LOW○○○ | RoB,Indirectnessx2, Imprecisionx2, Intransitivity |
| Sotagliflozin 200mg v.s Dulaglutide 1.5mg | 1.07 | 0.28 | 4.03 | VERY LOW○○○ | RoB,Indirectness, Imprecisionx2, Intransitivity |
| Sotagliflozin 200mg v.s Efpeglenatide 4 + 6mg | 0.82 | 0.21 | 3.11 | VERY LOW○○○ | RoB, Imprecisionx2, Intransitivity |
| Sotagliflozin 200mg v.s Empagliflozin | 0.81 | 0.21 | 3.11 | VERY LOW○○○ | RoB,Indirectness, Imprecisionx2, Intransitivity |
| Sotagliflozin 200mg v.s Ertugliflozin 5mg | 0.89 | 0.22 | 3.6 | VERY LOW○○○ | RoB, Imprecisionx2 |
| Sotagliflozin 200mg v.s Ertugliflozin 15mg | 1.1 | 0.27 | 4.44 | VERY LOW○○○ | RoB, Imprecisionx2 |
| Sotagliflozin 200mg v.s Exenatide | 1.07 | 0.27 | 4.21 | VERY LOW○○○ | RoB,Indirectness, Imprecisionx2, Intransitivity |
| Sotagliflozin 200mg v.s Insulin Glargine | 2.35 | 0.5 | 11.01 | VERY LOW○○○ | RoB,Indirectnessx2, Imprecisionx2, Intransitivity |
| Sotagliflozin 200mg v.s Linagliptin | 1.33 | 0.35 | 5.08 | VERY LOW○○○ | RoB, Imprecisionx2 |
| Sotagliflozin 400mg v.s Canagliflozin 100mg | 0.89 | 0.62 | 1.29 | LOW  ○○⊕ | RoB, Intransitivity |
| Sotagliflozin 400mg v.s Dapagliflozin 10mg | 0.77 | 0.52 | 1.14 | LOW  ○○⊕ | RoB, Intransitivity |
| Sotagliflozin 400mg v.s Dulaglutide 0.75mg | 1.99 | 0.83 | 4.78 | VERY LOW○○○ | RoB,Imprecisionx2, Intransitivityx2 |
| Sotagliflozin 400mg v.s Dulaglutide 1.5mg | 1.19 | 0.87 | 1.63 | VERY LOW○○○ | RoB,Indirectness, Intransitivityx2 |
| Sotagliflozin 400mg v.s Efpeglenatide 4 + 6mg | 0.91 | 0.65 | 1.29 | VERY LOW○○○ | RoB, Intransitivityx2 |
| Sotagliflozin 400mg v.s Empagliflozin | 0.91 | 0.64 | 1.3 | VERY LOW○○○ | RoB,Indirectness, Intransitivityx2 |
| Sotagliflozin 400mg v.s Ertugliflozin 5mg | 1 | 0.59 | 1.69 | VERY LOW○○○ | RoB,Imprecisionx2, Intransitivity |
| Sotagliflozin 400mg v.s Ertugliflozin 15mg | 1.23 | 0.73 | 2.08 | VERY LOW○○○ | RoB,Imprecisionx2, Intransitivity |
| Sotagliflozin 400mg v.s Exenatide | 1.2 | 0.76 | 1.89 | VERY LOW○○○ | RoB,Indirectness, Intransitivityx2 |
| Sotagliflozin 400mg v.s Insulin Glargine | 2.63 | 1.14 | 6.11 | VERY LOW○○○ | RoB,Indirectnessx2, Imprecisionx2, Intransitivityx2 |
| Sotagliflozin 400mg v.s Linagliptin | 1.49 | 1.07 | 2.09 | LOW  ○○⊕ | RoB, Intransitivity |
| Canagliflozin 100mg v.s Dapagliflozin 10mg | 1.16 | 0.82 | 1.64 | HIGH⊕⊕⊕ |  |
| Canagliflozin 100mg v.s Dulaglutide 0.75mg | 0.45 | 0.19 | 1.05 | VERY LOW○○○ | RoB,Indirectnessx2, Imprecisionx2, Intransitivityx2 |
| Canagliflozin 100mg v.s Dulaglutide 1.5mg | 0.75 | 0.58 | 0.96 | LOW  ○○⊕ | Indirectness, Intransitivity |
| Canagliflozin 100mg v.s Efpeglenatide 4 + 6mg | 0.98 | 0.73 | 1.3 | MODERATE○⊕⊕ | Intransitivity |
| Canagliflozin 100mg v.s Empagliflozin | 0.98 | 0.72 | 1.33 | LOW  ○○⊕ | Indirectness, Intransitivity |
| Canagliflozin 100mg v.s Ertugliflozin 5mg | 0.9 | 0.55 | 1.47 | HIGH⊕⊕⊕ |  |
| Canagliflozin 100mg v.s Ertugliflozin 15mg | 0.72 | 0.45 | 1.18 | HIGH⊕⊕⊕ |  |
| Canagliflozin 100mg v.s Exenatide | 0.75 | 0.49 | 1.13 | LOW | Indirectness, Intransitivity |
| Canagliflozin 100mg v.s Insulin Glargine | 0.34 | 0.15 | 0.77 | VERY LOW○○○ | Imprecisionx2, Intransitivity |
| Canagliflozin 100mg v.s Linagliptin | 0.6 | 0.45 | 0.79 | HIGH⊕⊕⊕ |  |
| Dapagliflozin 10mg v.s Dulaglutide 0.75mg | 0.39 | 0.16 | 0.92 | VERY LOW○○○ | Imprecisionx2, Intransitivity |
| Dapagliflozin 10mg v.s Dulaglutide 1.5mg | 0.65 | 0.49 | 0.86 | LOW  ○○⊕ | Indirectness, Intransitivity |
| Dapagliflozin 10mg v.s Efpeglenatide 4 + 6mg | 0.84 | 0.61 | 1.15 | MODERATE○⊕⊕ | Intransitivity |
| Dapagliflozin 10mg v.s Empagliflozin | 0.84 | 0.61 | 1.18 | LOW  ○○⊕ | Indirectness, Intransitivity |
| Dapagliflozin 10mg v.s Ertugliflozin 5mg | 0.77 | 0.46 | 1.29 | HIGH⊕⊕⊕ |  |
| Dapagliflozin 10mg v.s Ertugliflozin 15mg | 0.62 | 0.38 | 1.03 | HIGH⊕⊕⊕ |  |
| Dapagliflozin 10mg v.s Exenatide | 0.64 | 0.42 | 0.99 | LOW  ○○⊕ | Indirectness, Intransitivityx2 |
| Dapagliflozin 10mg v.s Insulin Glargine | 0.29 | 0.13 | 0.67 | VERY LOW○○○ | RoB,Indirectnessx2, Imprecisionx2, Intransitivityx2 |
| Dapagliflozin 10mg v.s Linagliptin | 0.51 | 0.38 | 0.7 | HIGH⊕⊕⊕ |  |
| Dulaglutide 0.75mg v.s Dulaglutide 1.5mg | 1.67 | 0.74 | 3.79 | VERY LOW○○○ | RoB,Indirectnessx2, Imprecisionx2 |
| Dulaglutide 0.75mg v.s Efpeglenatide 4 + 6mg | 2.18 | 0.94 | 5.06 | VERY LOW○○○ | RoB,Indirectnessx2, Imprecisionx2, Intransitivityx2 |
| Dulaglutide 0.75mg v.s Empagliflozin | 2.19 | 0.94 | 5.12 | VERY LOW○○○ | RoB,Indirectnessx2, Imprecisionx2, Intransitivityx2 |
| Dulaglutide 0.75mg v.s Ertugliflozin 5mg | 2 | 0.79 | 5.11 | VERY LOW○○○ | RoB,Indirectnessx2, Imprecisionx2, Intransitivityx2 |
| Dulaglutide 0.75mg v.s Ertugliflozin 15mg | 1.62 | 0.64 | 4.1 | VERY LOW○○○ | RoB,Indirectnessx2, Imprecisionx2, Intransitivityx2 |
| Dulaglutide 0.75mg v.s Exenatide | 1.67 | 0.68 | 4.07 | VERY LOW○○○ | RoB,Indirectnessx2, Imprecisionx2, Intransitivityx2 |
| Dulaglutide 0.75mg v.s Insulin Glargine | 0.76 | 0.38 | 1.5 | VERY LOW○○○ | RoB,Indirectnessx2, Imprecisionx2 |
| Dulaglutide 0.75mg v.s Linagliptin | 1.34 | 0.58 | 3.09 | VERY LOW○○○ | RoB,Indirectnessx2, Imprecisionx2, Intransitivityx2 |
| Dulaglutide 1.5mg v.s Empagliflozin | 1.31 | 1.04 | 1.65 | VERY LOW○○○ | Indirectness, Intransitivityx2 |
| Dulaglutide 1.5mg v.s Ertugliflozin 5mg | 1.2 | 0.76 | 1.89 | LOW  ○○⊕ | Indirectness, Intransitivity |
| Dulaglutide 1.5mg v.s Ertugliflozin 15mg | 0.97 | 0.62 | 1.51 | LOW  ○○⊕ | Indirectness, Intransitivity |
| Dulaglutide 1.5mg v.s Linagliptin | 0.8 | 0.66 | 0.97 | LOW  ○○⊕ | Indirectness, Intransitivity |
| Efpeglenatide 4 + 6mg v.s Empagliflozin | 1 | 0.77 | 1.32 | VERY LOW○○○ | Indirectness, Intransitivityx2 |
| Efpeglenatide 4 + 6mg v.s Ertugliflozin 5mg | 0.92 | 0.57 | 1.48 | MODERATE○⊕⊕ | Intransitivity |
| Efpeglenatide 4 + 6mg v.s Ertugliflozin 15mg | 0.74 | 0.47 | 1.18 | MODERATE○⊕⊕ | Intransitivity |
| Efpeglenatide 4 + 6mg v.s Exenatide | 0.76 | 0.52 | 1.13 | VERY LOW○○○ | Indirectness, Intransitivityx2 |
| Efpeglenatide 4 + 6mg v.s Insulin Glargine | 0.35 | 0.15 | 0.78 | VERY LOW○○○ | RoB,Indirectnessx2, Imprecisionx2, Intransitivityx2 |
| Efpeglenatide 4 + 6mg v.s Linagliptin | 0.61 | 0.48 | 0.78 | MODERATE○⊕⊕ | Intransitivity |
| Empagliflozin v.s Ertugliflozin 5mg | 0.92 | 0.56 | 1.49 | LOW  ○○⊕ | Indirectness, Intransitivity |
| Empagliflozin v.s Ertugliflozin 15mg | 0.74 | 0.46 | 1.19 | LOW  ○○⊕ | Indirectness, Intransitivity |
| Empagliflozin v.s Exenatide | 0.76 | 0.51 | 1.14 | VERY LOW○○○ | Indirectness, Intransitivityx2 |
| Empagliflozin v.s Insulin Glargine | 0.35 | 0.15 | 0.78 | VERY LOW○○○ | RoB,Indirectnessx2, Imprecisionx2, Intransitivityx2 |
| Empagliflozin v.s Linagliptin | 0.61 | 0.47 | 0.79 | LOW  ○○⊕ | Indirectness, Intransitivity |
| Ertugliflozin 5mg v.s Exenatide | 0.83 | 0.47 | 1.46 | VERY LOW○○○ | Indirectness,Imprecisionx2, Intransitivity |
| Ertugliflozin 5mg v.s Insulin Glargine | 0.38 | 0.15 | 0.93 | VERY LOW○○○ | RoB,Indirectnessx2, Imprecisionx2, Intransitivity |
| Ertugliflozin 5mg v.s Linagliptin | 0.67 | 0.42 | 1.07 | HIGH⊕⊕⊕ |  |
| Ertugliflozin 15mg v.s Exenatide | 1.03 | 0.59 | 1.79 | VERY LOW○○○ | Indirectness,Imprecisionx2, Intransitivity |
| Ertugliflozin 15mg v.s Insulin Glargine | 0.47 | 0.19 | 1.15 | VERY LOW○○○ | RoB,Indirectnessx2, Imprecisionx2, Intransitivity |
| Ertugliflozin 15mg v.s Linagliptin | 0.83 | 0.52 | 1.31 | HIGH⊕⊕⊕ |  |
| Exenatide v.s Insulin Glargine | 0.45 | 0.19 | 1.08 | VERY LOW○○○ | RoB,Indirectnessx2, Imprecisionx2, Intransitivityx2 |
| Exenatide v.s Linagliptin | 0.8 | 0.55 | 1.18 | LOW  ○○⊕ | Indirectness, Intransitivity |
| Insulin Glargine v.s Linagliptin | 1.76 | 0.79 | 3.94 | VERY LOW○○○ | Imprecisionx2, Intransitivityx2 |

GRADE, RoB, risk of bias; CI, confidence interval; GRADE, Grading of Recommendations Assessment, Development, and Evaluation

Table S4: GRADE evidence profile for eGFR outcomes in patients with T2DM and CKD

| Comparison | Network estimate | | | Final rating | Reasons for downgrading |
| --- | --- | --- | --- | --- | --- |
|  | Realtive estimate | | |  |  |
|  | Point estimate | CI lower limit | CI upper limit |  |  |
| Canagliflozin 300mg vs. Placebo | -4.98 | -10.36 | 0.40 | MODERATE  ○⊕⊕ | RoB |
| Canagliflozin 300mg vs. Canagliflozin 100mg | -6.69 | -12.05 | -1.33 | LOW  ○○⊕ | RoB, Inconsistency |
| Canagliflozin 100mg vs. Placebo | 1.71 | -0.31 | 3.72 | VERY LOW  ○○○ | RoB, Indirectnessx2 |
| Sotagliflozin 400mg vs. Placebo | -0.89 | -2.28 | 0.51 | MODERATE  ○⊕⊕ | RoB |
| Sotagliflozin 400mg vs. Sotagliflozin 200mg | -0.33 | -1.90 | 1.24 | MODERATE  ○⊕⊕ | RoB |
| Sotagliflozin 200mg vs. Placebo | -0.56 | -1.95 | 0.84 | MODERATE  ○⊕⊕ | RoB |
| Empegliflozin vs. Placebo | 0.90 | -0.42 | 2.22 | MODERATE  ○⊕⊕ | Indirectness |
| Semaglutide vs. Placebo | 1.17 | -2.50 | 0.16 | HIGH  ⊕⊕⊕ |  |
| Canagliflozin 300mg vs. Sotagliflozin 400mg | -4.10 | -9.65 | 1.46 | LOW  ○○⊕ | RoB, Intransitivity |
| Canagliflozin 300mg vs. Sotagliflozin 200mg | -4.43 | -9.98 | 1.13 | LOW  ○○⊕ | RoB, Intransitivity |
| Canagliflozin 300mg vs. Empegliflozin | -5.88 | -11.42 | -0.34 | LOW  ○○⊕ | RoB, Indirectness, Intransitivity |
| Canagliflozin 300mg vs. Semaglutide | -6.15 | -11.69 | -0.61 | LOW  ○○⊕ | RoB, Intransitivity |
| Canagliflozin 100mg vs. Sotagliflozin 400mg | 2.59 | 0.14 | 5.04 | VERY LOW  ○○○ | RoB,Indirectnessx2, Imprecisionx3, Intransitivity |
| Canagliflozin 100mg vs. Sotagliflozin 200mg | 2.26 | -0.19 | 4.71 | VERY LOW  ○○○ | RoB,Indirectnessx2, Intransitivity |
| Canagliflozin 100mg vs. Empegliflozin | 0.81 | -1.6 | 3.22 | VERY LOW  ○○○ | RoB,Indirectnessx2, Intransitivity |
| Canagliflozin 100mg vs. Semaglutide | 0.54 | -1.88 | 2.95 | VERY LOW  ○○○ | RoB, Indirectnessx2 |
| Sotagliflozin 400mg vs. Empegliflozin | -1.79 | -3.71 | 0.14 | LOW  ○○⊕ | RoB, Indirectness, Intransitivity |
| Sotagliflozin 400mg vs. Semaglutide | -2.06 | -3.99 | -0.13 | LOW  ○○⊕ | RoB, Intransitivity |
| Sotagliflozin 200mg vs. Empegliflozin | -1.46 | -3.38 | 0.47 | LOW  ○○⊕ | RoB, Indirectness, Intransitivity |
| Sotagliflozin 200mg vs. Semaglutide | -1.73 | -3.66 | 0.2 | LOW  ○○⊕ | RoB, Intransitivity |
| Empegliflozin vs. Semaglutide | -0.27 | -2.14 | 1.6 | LOW  ○○⊕ | Indirectness, Intransitivity |

RoB, risk of bias; GRAD, Grading of Recommendations Assessment, Development, and Evaluation; CI, confidence interval

Table S5. GRADE evidence profile for UACR outcomes in patients with T2DM and CKD

| **Comparison** | **Network estimate** | | | **Final rating** | **Reasons for downgrading** |
| --- | --- | --- | --- | --- | --- |
|  | **Relative estimate** | | |  |  |
|  | **Point estimate** | **CI lower limit** | **CI upper limit** |  |  |
| Canagliflozin 300 mg vs. Canagliflozin 100 mg | 0.968 | 0.708 | 1.322 | MODERATE ○⊕⊕ | RoB |
| Canagliflozin 300 mg vs. Placebo | 1.747 | 1.289 | 2.367 | MODERATE ○⊕⊕ | RoB |
| Canagliflozin 100 mg vs. Placebo | 1.690 | 1.364 | 2.095 | VERY LOW ○○○ | RoB, Indirectness x2 |
| Dapagliflozin 10 mg vs. Placebo | 0.800 | 0.607 | 1.054 | HIGH ⊕⊕⊕ |  |
| Canagliflozin 300 mg vs. Dapagliflozin 10 mg | 0.716 | 0.475 | 1.079 | MODERATE ○⊕⊕ | RoB |
| Canagliflozin 100 mg vs. Dapagliflozin 10 mg | 0.740 | 0.522 | 1.050 | VERY LOW ○○○ | RoB, Indirectness x2, Intransitivity |

Rob, risk of bias; GRADE, Grading of Recommendations Assessment, Development, and Evaluation; UACR, urine albumin-creatinine ratio

Table S6. P score of renal composite outcome

|  | **P-score (random)** |
| --- | --- |
| Dapagliflozin 10 mg | 0.9205 |
| Canagliflozin 100 mg | 0.8019 |
| Empagliflozin | 0.782 |
| Ertugliflozin 5 mg | 0.6817 |
| Sotagliflozin 400 mg | 0.6456 |
| Dulaglutide 1.5 mg | 0.4976 |
| Exenatide | 0.4866 |
| Sotagliflozin 200 mg | 0.455 |
| Ertugliflozin 15 mg | 0.4529 |
| Placebo | 0.3047 |
| Linagliptin | 0.2292 |
| Dulaglutide 0.75 mg | 0.1818 |
| Insulin Glargine | 0.0602 |
